# Characterization of OxyR as a Negative Transcriptional Regulator That Represses Catalase Production in *Corynebacterium diphtheriae*

**DOI:** 10.1371/journal.pone.0031709

**Published:** 2012-03-16

**Authors:** Ju-Sim Kim, Randall K. Holmes

**Affiliations:** Dept of Microbiology, University of Colorado School of Medicine, Aurora, Colorado, United States of America; The Methodist Hospital Research Institute, United States of America

## Abstract

*Corynebacterium diphtheriae* and *Corynebacterium glutamicum* each have one gene (*cat*) encoding catalase. In-frame Δ*cat* mutants of *C. diphtheriae* and *C. glutamicum* were hyper-sensitive to growth inhibition and killing by H_2_O_2_. In *C. diphtheriae* C7(β), both catalase activity and *cat* transcription decreased ∼2-fold during transition from exponential growth to early stationary phase. Prototypic OxyR in *Escherichia coli* senses oxidative stress and it activates *katG* transcription and catalase production in response to H_2_O_2_. In contrast, exposure of *C. diphtheriae* C7(β) to H_2_O_2_ did not stimulate transcription of *cat*. OxyR from *C. diphtheriae* and *C. glutamicum* have 52% similarity with *E. coli* OxyR and contain homologs of the two cysteine residues involved in H_2_O_2_ sensing by *E. coli* OxyR. In-frame Δ*oxyR* deletion mutants of *C. diphtheriae* C7(β), *C. diphtheriae* NCTC13129, and *C. glutamicum* were much more resistant than their parental wild type strains to growth inhibition by H_2_O_2_. In the *C. diphtheriae* C7(β) Δ*oxyR* mutant, *cat* transcripts were about 8-fold more abundant and catalase activity was about 20-fold greater than in the C7(β) wild type strain. The *oxyR* gene from *C. diphtheriae* or *C. glutamicum*, but not from *E. coli*, complemented the defect in Δ*oxyR* mutants of *C. diphtheriae* and *C. glutamicum* and decreased their H_2_O_2_ resistance to the level of their parental strains. Gel-mobility shift, DNaseI footprint, and primer extension assays showed that purified OxyR from *C. diphtheriae* C7(β) bound, in the presence or absence of DTT, to a sequence in the *cat* promoter region that extends from nucleotide position −55 to −10 with respect to the +1 nucleotide in the *cat* ORF. These results demonstrate that OxyR from *C. diphtheriae* or *C. glutamicum* functions as a transcriptional repressor of the *cat* gene by a mechanism that is independent of oxidative stress induced by H_2_O_2_.

## Introduction

Aerobic organisms have specific mechanisms to protect themselves from reactive oxygen species (ROS) such as superoxide radical (O^−^_2_) and hydrogen peroxide (H_2_O_2_), which can be generated by incomplete reduction of O_2_ during respiration. The Fenton reaction, which generates hydroxyl radicals by reduction and oxidation of Fe ions in the presence of ROS, has been proposed as a mechanism for cell damage [1]. The ROS may cause oxidative damage to DNA, protein, and lipid [2]. Major defense systems against ROS often involve superoxide dismutase (SOD), catalase, and peroxidase, which function cooperatively to convert the ROS into O_2_ and H_2_O.

*Escherichia coli* produces two types of catalase, both of which can detoxify H_2_O_2_ by converting it to H_2_O and O_2_. The first is a bifunctional catalase hydroperoxidase I (HPI) encoded by *katG* that has both catalase and peroxidase activities, and the *katG* gene is transcriptionally activated by the positive regulator OxyR [3]. A monofunctional HPII encoded by *katE* has catalase activity only, and *katE* is activated during stationary phase [3] but is not induced by OxyR [4].

The two best characterized transcriptional regulators that mediate responses to oxidative stress in *E. coli* are SoxRS and OxyR. SoxR is a transcriptional activator with an iron-sulfur cluster that can be oxidized to the [2Fe-2S] state by exposure to the superoxide radical or nitric oxide. Both the reduced and the oxidized forms of SoxR can bind to its DNA target sequence [5], [6], but only the oxidized form of SoxR increases the transcription of *soxS*. The SoxS protein then activates transcription of *sodA* (superoxide dismutase), *zwf* (glucose-6-phosphate dehydrogenase), *fpr* (NADPH-ferredoxin reductase), *nfo* (DNA repair endonuclease IV), and *acrAB* (an efflux pump) in *E. coli* [7], [8].

The other major redox-sensing protein from *E. coli*, OxyR, functions both as a hydrogen peroxide sensor and as a transcriptional activator. *E.coli* OxyR, a member of the LysR family of transcription factors [9], has a helix-turn-helix (HTH) DNA binding motif in its N-terminal region and regulatory and oligomerization motifs in its C-terminal region. Oxidation of OxyR by hydrogen peroxide converts two cysteine residues (Cys199 and Cys208) to a disulfide bond, and the oxidized form of OxyR can then bind to specific DNA sequences in the regulatory regions of target genes [10]. The OxyR-activated genes in *E.coli* include *dps* (a DNA-binding protein from starved cells), *gorA* (GSH reductase), *grxA* (glutaredoxin), *katG* (calatase/peroxidase), *ahpCF* (alkyl hydroperoxide-NADPH oxidoreductase), and *fur* (an iron-binding repressor of iron transport) [7], [11], [12]. Products of genes in the OxyR regulon have many important functions in antioxidant defenses, including degradation of hydrogen peroxide by catalase and protection of DNA from oxidative attack by Dps proteins. In addition, *E.coli* OxyR activates production of OxyS, a small protein that regulates as many as 20 additional gene products [13].

Recent studies in *Neisseria gonorrhoeae* and *Neisseria meningitidis* indicate that OxyR can function both as a repressor of catalase gene expression in the absence of oxidative stress and as an activator of catalase gene expression in response to H_2_O_2_. In an *oxyR* mutant of *N. gonorrhoeae*, the basal level of catalase activity was greatly increased, and induction of catalase by hydrogen peroxide was abolished [14], [15]. In wild-type *N. meningitidis*, transcription of the catalase gene was activated in response to H_2_O_2_ in an OxyR-dependent manner, but transcription of the catalase gene in an *oxyR* null mutant was at a constitutive intermediate level between the un-induced and H_2_O_2_–induced levels in the wild type strain [16].

*Corynebacterium diphtheriae* is gram-positive, non-spore-forming aerobic bacterium that produces diphtheria toxin and causes the severe respiratory disease diphtheria [17]. The diphtheria toxin repressor (DtxR) in *C. diphtheriae* represses diphtheria toxin production and iron uptake in response to high-iron conditions [18], [19], [20]. A *C. diphtheriae* C7(β) Δ*dtxR* mutant exhibited increased susceptibility to growth inhibition and killing in response to oxidative stress [21]. Only a few studies other studies of oxidative stress responses have been reported in bacteria from the genus *Corynebacterium*. Merkamm and Guyonvarch cloned the *sodA* gene from *C. meiassecola* and reported that superoxide dismutase played a role in cell viability [22]. The *sodA* gene was also cloned from *C. glutamcium* and shown to encode a strictly manganese-dependent form of superoxide dismutase [23].

In this study, we investigated the roles of the *cat* and *oxyR* genes of *C. diphtheriae* and *C. glutamicum* in the responses of these bacteria to H_2_O_2_-induced oxidative stress. We also characterized the effects of growth conditions on *cat* expression and showed that OxyR in *C. diphtheriae* functions as a repressor, but not as an activator, of *cat* by a mechanism that is independent of H_2_O_2_-induced oxidative stress.

## Methods

### Bacterial stains, plasmids, and growth conditions

The bacterial strains used in this study are listed in Table 1, and the plasmids used in this study are listed in Table 2. Strains of *C. diphtheriae* and *C. glutamicum* were grown at 37°C and 30°C, respectively, in heart infusion broth (Difco, Detroit, Mich.) containing 0.2% Tween 80 (designated HIBTW) or in PGT medium [24] as described for specific experiments. Low-iron PGT medium was deferrated by treatment with Chelex resin (Bio-Rad, Hercules, CA) [25], and high-iron PGT medium was made by adding 10 µM ferric chloride to the low-iron PGT medium. Exponential phase cultures of *C. diphtheriae* were typically harvested when A600 was between 0.8 and 1.0, and early stationary phase cultures of *C. diptherheriae* were typically harvested when A600 was between 6 and 7. Transconjugants of *C. diphtheriae* and *C. glutamicum* were selected on HIBTW agar plates containing 50 µg/ml of kanamycin and 30 µg/ml of nalidixic acid [21]. Strains of *E. coli* were grown at 37°C in Luria-Bertani (LB) medium [26] to which ampicillin (Ap), chloramphenicol (Cm), kanamycin (Km), or tetracycline (Tc) was added at a final concentration of 50, 20, 25, or 10 µg/ml, respectively, when indicated. Growth of *C. diphtheriae* or *C. glutamicum* was measured by determining A600 for culture samples appropriately diluted in 1× PBS (phosphate buffered saline) [26].

**Table 1:** Bacteria used in this study.

Strains	Relevant characteristics	Reference or source
***Corynebacterium***		
*C. diphtheriae* C7(β)	wild-type reference strain, *tox* ^+^, lysogenic for phase β	[24]
*C. diphtheriae*	NCTC13129, wild-type strain	lab strain
*C. glutamicum*	ATCC13032, wild-type strain	lab strain
C7(β) Δ*cat*	*C. diphtheriae* C7(β), Δ*cat*	This study
C7(β) Δ*oxyR*	*C. diphtheriae* C7(β), Δ*oxyR*	This study
NCTC Δ*cat*	*C. diphtheriae* NCTC13129, Δ*cat*	This study
NCTC Δ*oxyR*	*C. diphtheriae* NCTC13129, Δ*oxyR*	This study
Cg Δ*cat*	*C. glutamicum* ATCC13032, Δ*cat*	This study
Cg Δ*oxyR*	*C. glutamicum* ATCC13032, Δ*oxyR*	This study
***E.coli***		
DH5α	*supE*44 Δ*lacU*169 (φ80 *lacZ* ΔM15) *hsdR*17 *recA*1	[50]
	*endA*1 *gyrA*96 *thi*-1 *relA*1	
S17-1	C600::RP4 2-(Tc::Mu)(Km::Tn7) *thi pro hsdR hsdM* ^+^ *recA*	[28]
GC4468	Δ*lacU*169 *rpsL*	[41]
JL102	GC4468, *oxyR* mutant; Km^r^	[41]
OrigamiB(DE3)	*E. coli* K12 F- *ompT hsdSB*(rB- mB-) *gal dcm lacY*1 *aphC*	Novagen
pLysS	(DE3) *gor*522::Tn10 *trxB* pLysS; Cm^r^, Km^r^, Tc^r^	

**Table 2:** Plasmids used in this study.

Plasmids	Relevant characteristics	Reference or source
pCR2.1 TOPO	pUC *ori*, cloning for PCR DNA fragment; Ap^r^	Invitrogen
pET-22b (+)	*ori* pBR322, C-terminal His6-Taq fusion vector; Ap^r^	Novagen
pET-Eco OxyR	pET-22b (+)+0.9-kb DNA containing *oxyR* of GC4468; Ap^r^	This study
pET-OxyR	pET-22b (+)+0.9-kb DNA containing *oxyR* of C7(β); Ap^r^	This study
pK19*mobsacB*	*ori* T of RP4 *sacB*; Km^r^	[27]
pK-Δ*oxyR*	pK19*mobsacB*+1.6-kb DNA containing in-frame deleted *oxyR* of	This study
	of C7(β); Km^r^	
pK-Δ*cat*	pK19*mobsacB*+2.0-kb DNA containing in-frame deleted *cat* of	This study
	C7(β); Km^r^	
pK-Δ*oxyR*_NCTC_	pK19*mobsacB*+1.6-kb DNA containing in-frame deleted *oxyR* of	This study
	NCTC13129; Km^r^	
pK-Δ*cat*_NCTC_	pK19*mobsacB*+2.0-kb DNA containing in-frame deleted *cat* of	This study
	NCTC13129; Km^r^	
pK-Δ*oxyR*_Cg_	pK19*mobsacB*+2.0-kb DNA containing in-frame deleted *oxyR* of	This study
	*C. glutamicum* ATCC13032; Km^r^	
pK-Δ*cat*_Cg_	pK19*mobsacB*+2.0-kb DNA containing in-frame deleted *cat* of	This study
	*C. glutamicum* ATCC13032; Km^r^	
pK-PIM	*ori* E of pUC19, *ori* T of RP4, *attB attP* phase *int*, site-specific	[30]
	integrated plasmid; Km^r^	
pK-PIM-*oxyR* C7(β)	pK-PIM+2.0-kb DNA containing *oxyR* of C7(β); Km^r^	This study
pK-PIM-*oxyR* Eco	pK-PIM+1.1-kb DNA containing *oxyR* of GC4468; Km^r^	This study
pK-PIM-*oxyR* Eco2	pK-PIM+0.5-kb p*oxyR* DNA of C7(β)+0.9-kb *oxyR* ORF of	This study
	GC4468; Km^r^	
pSPZ	promoterless *lacZ* in shuttle vector for *C. diphtheriae*, pJKS1; Sp^r^	[31]
pKPL	pK-PIM with promoterless *lacZ*; Km^r^	This study
pPLΩ	pK-PIM+Ω::*lacZ* fusion; Sm^r^/Sp^r^, Km^r^	This study
pPL-*cat*100	pK-PIM+*cat::lacZ* fusion; 100-bp fragment of *cat* start codon;	This study
	Sm^r^/Sp^r^, Km^r^	
pPL-*cat*200	pK-PIM+*cat::lacZ* fusion; 200-bp fragment of *cat* start codon;	This study
	Sm^r^/Sp^r^, Km^r^	
pPL-*cat*300	pK-PIM+*cat::lacZ* fusion; 300-bp fragment of *cat* start codon;	This study
	Sm^r^/Sp^r^, Km^r^	
pRK415	*ori* IncP Mob RP4 *lacZα*; Tc^r^	[42]
pRK-*oxyR* Eco	pRK415+1.1-kb DNA containing *oxyR* of GC4468; Tc^r^	This study
pRK-*oxyR* Eco2	pRK415+0.5-kb p*oxyR* DNA of C7(β)+0.9-kb *oxyR* ORF of	This study
	GC4468; Tc^r^	
pT-*cat* A	pCR2.1 TOTO+110-bp PCR DNA fragment with *cat*-F12+*cat*-R9	This study
	primers; Ap^r^	
pT-*cat* B	pCR2.1 TOTO+110-bp PCR DNA fragment with *cat*-F13+*cat*-R10	This study
	primers; Ap^r^	
pT-*cat* C	pCR2.1 TOTO+110-bp PCR DNA fragment with *cat*-F14+*cat*-R11	This study
	primers; Ap^r^	
pT-*cat* foot	pCR2.1 TOTO+244-bp PCR DNA fragment with *cat*-F13+*cat*-R12	This study
	primers; Ap^r^	

### Construction of in-frame deletion mutations in *C. diphtheriae* and *C. glutamicum*

(i) Construction of Δ*oxyR* allele for *C. diphtheriae* C7(β). A 789-bp upstream DNA fragment extending from nucleotide position −685 to 104 relative to the *oxyR* initiation codon was amplified from *C. diphtheriae* C7(β) genomic DNA by PCR using the forward primer *oxyR*-F1 (5′-CCGCAT**CTGCAG**TAACAG-3′, with the underlined mutation to introduce the *Pst*I site shown in bold font) and the reverse primer *oxyR*-R1 (5′-GAAATCGA**AAGCTT**GGCAGC-3′, containing the *Hin*dIII site indicated in bold font). A 869-bp downstream DNA fragment from nucleotide position −39 to 830 relative to the OxyR stop codon was amplified with the forward primer *oxyR*-F2 (5′-GTTGGCCAA**AAGCTT**CAAGAC-3′, with the underlined mutations to introduce the *Hin*dIII site shown in bold font) and the reverse primer *oxyR*-R2 (5′-CCATAAAGATCT**TCTAGA**TCCG-3′, with the underlined mutations to introduce the *Xba*I site shown in bold font). The 789-bp *Pst*I/*Hin*dIII-cut upstream DNA fragment and 869-bp *Hin*dIII/*Xba*I-cut downstream DNA fragment were cloned into *Pst*I/*Xba*I-cut pK19*mobsacB* [27] to generate pK-Δ*oxyR*.

(ii) Construction of Δ*cat* allele *C. diphtheriae* C7(β). A 1050-bp upstream DNA fragment extending from nucleotide position −1072 to +33 relative to the *cat* initiation codon was PCR amplified from *C. diphtheriae* C7(β) genomic DNA by PCR using the forward primer *cat*-F3 (5′-GCCGCCAAA**AAGCTT**ATCGAG-3′, with the underlined mutations to introduce the *Hin*dIII site shown in bold font) and the reverse primer *cat*-R1 (5′-CTTGTTCA**GAATTC**GATCGAC-3′, with the underlined mutations to introduce the *Eco*RI site shown in bold font). A 922-bp downstream DNA fragment extending from −22 to +900 relative to the *cat* stop codon was amplified from *C. diphtheriae* C7(β) genomic DNA by PCR using the forward primer *cat*-F2 (5′-GTGCGA**GAATTC**GTGAGGCATT-3′, with the underlined mutations to introduce the *Eco*RI site shown in bold font) and the reverse primer *cat*-R2 (5′-GCAAGGTGT**TCTAGA**GGTCG-3′, with the underlined mutations to introduce the *Xba*I site shown in bold font). The 1050 bp *Hin*dIII/*Eco*RI-cut upstream DNA fragment and the 922 bp *Eco*RI/*Xba*I-cut downstream fragment were cloned into *Hin*dIII/*Xba*I-cut pK19*mobsacB* [27] to generate pK-Δ*cat*.

(iii) Construction of Δ*oxyR* allele for *C. diphtheriae* NCTC13129. The *oxyR*-F1/*oxyR*-R1 and *oxyR*-F2/*oxyR*-R2 primer pairs described previously were used with *C. diphtheriae* NCTC13129 genomic DNA to PCR amplify 789-bp upstream and 869-bp downstream DNA sequences for the *oxyR* gene. The *Pst*I/*Hin*dIII-cut upstream DNA fragment and *Hin*dIII/*Xba*I-cut downstream DNA fragment were cloned into *Pst*I/*Xba*I-cut pK19*mobsacB* [27] to generate pK-Δ*oxyR*_NCTC_

(iv) Construction of Δ*cat* allele for *C. diphtheriae* NCTC13129. The *cat*-F3/*cat*-R1 and *cat*-F2/*cat*-R2 primer pairs described previously were used with *C. diphtheriae* NCTC13129 genomic DNA to PCR amplify 1045-bp upstream and 922-bp downstream DNA sequences for the *cat* gene. The *Pst*I/*Hin*dIII-cut upstream DNA fragment and *Hin*dIII/*Xba*I-cut downstream DNA fragment were cloned into *Pst*I/*Xba*I-cut pK19*mobsacB* [27] to generate pK-Δ*cat*_NCTC_.

(v) Construction of Δ*oxyR* allele for *C. glutamicum* ATCC13032. A 1015-bp DNA upstream DNA fragment extending from nucleotide position −974 to +41 relative to the *oxyR* initiation codon was amplified from *C. glutamicum* genomic DNA by PCR using the forward primer cg-*oxyR*-F1 (5′-CGGTGA**AAAGCTT**GAAGCTC-3′, with the underlined mutations to introduce the *Hin*dIII site shown in bold font) and the reverse primer cg-*oxyR*-R1 (5′-CGAAGCTG**CTCGAG**TGTG -3′ containing the underlined mutations to introduce the *Xho*I site indicated in bold font). A 961-bp DNA downstream DNA fragment extending from −25 to +936 relative to the *oxyR* stop codon was amplified with the forward primer cg-*oxyR*-F2 (5′-GCAAAAT**CTCGAG**GTAGCGCAG-3′, containing the underlined mutations to introduce the *Xho*I site shown in bold font) and the reverse primer cg-*oxyR*-R2 (5′-GTACCA**GAATTC**CTACCAAG-3′, containing the *Eco*RI site shown in bold font). The 1014-bp *Hin*dIII/*Xho*I-cut upstream DNA fragment and 960-bp *Xho*I/*Eco*RI-cut downstream DNA fragment were cloned into *Hin*dIII/*Eco*RI-cut pK19*mobsacB* [27] to generate pK-Δ*oxyR*_Cg_.

(vi) Construction of Δ*cat* allele for *C. glutamicum* ATCC13032. A 1036-bp upstream DNA fragment extending from nucleotide position −964 to +72 relative to the *cat* initiation codon was PCR amplified from *C. glutamicum* genomic DNA by PCR using forward primer cg-*cat*-F1 (5′-CTTCCATTG**CTGCAG**GAA-3′, with the underlined mutation to introduce the *Pst*I site shown in bold font) and cg-*cat*-R1-(5′-GTTTCCAGA**AAGCTT**TGGAC-3′) containing the *Hin*dIII site shown in bold font). A 1026-bp downstream flanking region extending from −34 to +992 relative to the *cat* stop codon was PCR amplified using the forward primer cg-*cat*-F2 (5′-CGTCAAG**AAGCTT**TACCTC-3′, with the underlined mutation to introduce the *Hin*dIII site shown in bold) and cg-*cat*-R2 (5′-CCTCCAG**TCTAGA**ATGAATC-3′, with the underlined mutations to introduce the *Xba*I site shown in bold). The 1036-bp *Pst*I/*Hin*dIII-cut upstream DNA fragment and the 1026-bp *Hin*dIII/*Xba*I-cut downstream DNA fragment were cloned into *Pst*I/*Xba*I-cut pK19*mobsacB* [27] to generate pK-Δ*cat*_Cg_.

(vii) The pK19*mobsac*B-derived plasmids described above were transformed into *E.coli* S17-1 [28] and mobilized by conjugation into *C. diphtheriae* C7(β), *C. diphtheriae* NCTC13129, or *C. glutamicum* ATCC13032, as appropriate. Kanamycin was used to select for co-integrates, and sucrose counter-selection was used to identify resolved co-integrates which were then screened to identify isolates carrying the desired Δ*oxyR* or Δ*cat* alleles. Details for these procedures were described previously [29].

### Agar diffusion growth inhibition assays for susceptibility to H_2_O_2_

Bacteria were cultured in low-iron PGT medium or PGT medium with 10 µM FeCl_3_, as indicated, and were harvested in exponential phase (A600 between 0.8 and 1.5). Samples containing approximately 10^7^ bacteria were spread on the surface of HIA agar plates. A 10 µl drop of 1 M H_2_O_2_ (for *C. diphtheriae* strains) or 4 M H_2_O_2_ (for *C. glutamicum* strains) was spotted onto the center of the plate, and the diameter of the growth inhibition zone was measured after overnight incubation.

### Construction and use of *cat::lacZ* reporter plasmids

*Not*I/*Xba*I-cut pK-PIM [30] was treated with the Klenow fragment of DNA polymerase and ligated with the blunt-ended 3.6-kb *Sma*I/*Xmn*I DNA fragment from pSPZ, which contains the *lacZ* gene [31], to generate pKPL. DNA fragments containing 127-bp, 227-bp, or 327-bp from the regulatory region upstream from the *cat* gene were amplified from chromosomal DNA of *C. diphtheriae* C7(β) by PCR using the forward primer *cat*-F5 (5′-**AAGCTT**AGGTAATACCTAACAAAA-3′, added *Hin*dIII sequence in bold font), *cat*-F6 (5′-**AAGCTT**GATCGCCACAGCCAGCC-3′, added *Hin*dIII sequence in bold font), and *cat*-F7 (5′-**AAGCTT**CTTGGGTGAGATCGTC-3′, added *Hin*dIII sequence in bold font), respectively, and the reverse primer *cat*-R5 (5′-CAGGATTG**TCTAGA**CTGGGC-3′, with the underlined mutations to introduce the *Xba*I site shown in bold font). The expected sequences of the PCR products were confirmed by DNA sequence analyses. Each *Hin*dIII/*Xba*I-cut PCR product was cloned, together with the *Spe*I/*Hind*III-cut 2.0 kb Ω DNA (Sm^r^/Sp^r^) [32], into *Spe*I/*Xba*I-cut pKPL to generate pPL-*cat*100, pPL-*cat*200, and pPL-*cat*300, respectively. Each of these plasmids contains transcription and translation stops in the upstream Ω DNA fragment that block any fortuitous transcriptional or translational read-through from the vector DNA into *cat*. Plasmid pPLΩ contains the Ω DNA upstream from the promoterless *lacZ* gene as a negative control. C. *diphtheriae* C7(β) cells harboring pPLΩ, pPL-*cat*100, pPL-*cat*200, or pPL-*cat*300 were grown in low-iron PGT medium or PGT medium with 10 µM FeCl_3_. β-galactosidase activity (Miller units) was determined in samples collected during logarithmic phase growth (A600 between 0.8 and 1.5) and in early stationary phase (A600 between 6 and 7) with o-nitrophenyl-β-galactoside substrate as described previously [33]. The enzyme activities were calculated as A420×1000/[time (min)×A600×cell volume (ml)] as described [33].

### Assays for catalase activity

Spectrophotometric assays for catalase activity were performed by monitoring the rate of degradation of H_2_O_2_ at A240 over 90 sec at room temperature. Each 1 ml reaction mixture contained 20 µg of crude soluble bacterial protein in 100 mM potassium phosphate, pH 7.4. The reactions were started by adding H_2_O_2_ to a final concentration of 10 mM. Catalase specific activity is expressed as units corresponding to 1 mmol of H_2_O_2_ decomposed/mg protein/min [34]. Staining of native polyacrylamide (12%) gels for catalase activity was performed as described previously [35]. Samples of crude bacterial extracts containing 20 µg of protein were electrophoretically separated on native polyacrylamide gels. Each gel was rinsed with distilled water and soaked for 10 min in 50 mM potassium phosphate buffer (pH 7.0) containing 10 mM H_2_O_2_. The gel was washed once again with distilled water, and then soaked for several minutes in 50 ml of a solution containing 1% ferric chloride and 1% potassium ferricyanide to allow the background color to develop. No color develops at the position(s) where catalase is present and H_2_O_2_ is degraded. Protein concentrations were determined by a BCA protein assay kit (Pierce, Rockford, IL) using bovine serum albumin (BSA) as the standard.

### RNA isolation and quantitative reverse-transcriptase PCR (qRT-PCR)

RNA isolation was performed as described previously [21]. To study effects of H_2_O_2_ on abundance of *cat* transcripts, *C. diphtheriae* C7(β) was grown in PGT medium with 10 µM FeCl_3_ to mid exponential phase (A600 between 2.5 and 3.0) and treated with H_2_O_2_ at a final concentration of 1 µM, 100 µM, 1 mM or 10 mM for 5 min or 30 min. To compare the abundance of *cat* transcripts in wild-type C7(β) and C7(β) Δ*oxyR* at different stages of growth, each bacterial strain was grown in low-iron PGT medium or PGT medium with 10 µM FeCl_3_ and harvested either during exponential phase (A600 between 0.8 and 1.5) or early stationary phase (A600 between 6 and 7). Cells were suspended in acid phenol and disrupted by shaking three times for 1 min for each treatment in a bead-beater. After centrifugation, RNA was extracted from the supernatant with acidified phenol/chlorophorm, precipitated with ethanol, and treated with RNase free-DNaseI (Promega, Madison, WI). RNA concentration was measured with a NanoDrop spectrophotometer (Thermo Scientific, Wilmington, DE).

Each cDNA synthesis reaction mixture contained 1 µg of purified bacterial RNA, Cepheid Omnimix bead (Takara, Japan), specific *cat* primers, and a *cat* probe with 5′ 6-carboxyfluorescein and 3′ black hole quencher 1 modifications. Reactions were performed in a Cepheid Smartcycler II (Cepheid, Sunnyvale, CA). Genomic DNA containing the gene of interest was used to generate a standard curve for each probe using identical experimental conditions. The abundance of the *cat* transcripts was normalized to the abundance *dnaE* transcripts for each RNA sample.

### Primer extension analysis of *cat* transcripts

Primer extension analysis of *cat* transcripts was performed with reverse primer *cat*-PE1 (5′-GTTCAGGATTGGATCGACTG-3′), an anti-sense sequence that pairs approximately with codons 4–10 of the *cat* transcript. The reverse primer was 5′-labeled with [γ-^32^P]ATP using T4 polynucleotide kinase (Promega, Madison, WI). Total RNA from *C. diphtheriae* C7(β) was purified from samples harvested in the exponential phase of growth (A600 between 0.8 and 1.5) and used as template for dideoxy-termination sequencing with the Thermo Sequenase™ cycle sequencing kit (Amersham pharmacia biotech, Cleveland, OH). The product of the primer extension reaction was analyzed on an 8.3 M urea-8% polyacrylamide sequencing gel, and the start site for the *cat* transcript was deduced from the experimentally determined sequence of the complementary primer extension product.

### Production and purification of recombinant *C. diphtheriae* OxyR and *E.coli* OxyR

For cloning into the C-terminal His6-fusion plasmid pET-22b (+) (Novagen, Madison, WI), the initiation and stop codons of the *oxyR* gene from *C. diphtheriae* C7(β) were modified to introduce *Nco*I and *Xho*I sites, respectively. The forward primer *oxyR*-F5 (5′-ATAAATAG**CCATGG**GCAATA-3′, with the underlined mutation in the *oxyR* start codon to introduce the *Nco*I site shown in bold font) was used in PCR with *C. diphtheriae* C7(β) genomic DNA as template and the reverse primer *oxyR*-R4 (5′-TTATGCTA**CTCGAG**CGCGAT-3′, with the underlined mutations in and near the OxyR stop codon to introduce the *Xho*I site shown in bold font). The *Nco*I/*Xho*I-cut PCR product was cloned into *Nco*I/*Xho*I-cut pET-22b (+) to generate pET-OxyR. Sequence analysis confirmed that the inserted fragment was in the correct reading frame and that no unexpected mutations were present in the amplified DNA.

*E. coli* Origami B (DE3) pLysS (Novagen, Madison, WI) containing pET-OxyR was grown in LB broth at 37°C to A600 between 0.5–0.7. Then IPTG was added at a final concentration of 0.1 mM to induce production of the C-terminally His6-tagged *C. diphtheriae* OxyR protein, and incubation was continued at room temperature. The bacteria were harvested 3 h after IPTG induction, disrupted by sonication, and centrifuged at 4°C to obtain cell-free supernatant. The His6-tagged fusion protein was purified from the cell lysate using TALON metal affinity resin (Clontech, Mountain View, CA) according to the manufacturer's protocol. The mass of the recombinant OxyR monomer was approximately 34 kDa, and the recombinant oligomeric OxyR protein was purified to apparent homogeneity as assessed by SDS-PAGE (12% polyacrylamide) (data not shown). The concentrations of purified OxyR used in gel-mobility shift assays and DNaseI footprinting assays were determined with the BCA protein assay kit (Pierce, Rockford, IL).

For cloning into pET-22b (+) (Novagen, Madison, WI), *Nde*I and *Xho*I sites were introduced at the initiation and stop codons of *E.coli oxyR*, respectively. The forward primer Eco *oxyR*-F3 (5′-GAGGATGG**CATATG**AATATTC-3′, with underlined mutations at the *oxyR* start codon to generate the *Nde*I site shown in bold font) was used with *E. coli* K12 genomic DNA as template and with the reverse primer Eco *oxyR*-R2 (5′-TTAAAC**CTCGAG**AACCGCCTGTTTTA-3′ containing the *Xho*I site overlapping the *oxyR* stop codon and shown in bold font) for PCR amplification of *E. coli oxyR*. The PCR product was digested with *Nde*I and *Xho*I and cloned into *Nde*I/*Xho*I-cut pET-22b (+) to generate pET-Eco OxyR. DNA sequence analysis confirmed the expected sequence and correct reading frame of the insert. The pET-Eco OxyR plasmid was introduced into *E. coli* Origami B (DE3) pLysS (Novagen. Madison, WI). This strain was grown at 37°C in LB medium, induced during exponential growth phase with IPTG, and the C-terminally His6-tagged *E. coli* OxyR protein was purified as described above for the comparable recombinant *C. diphtheriae* OxyR protein.

### Gel-mobility shift assays

The 110-bp overlapping DNA probes designated (a), (b), and (c) are *Eco*RI fragments from the pT-*cat* A, pT-*cat* B, and pT-*cat* C plasmids described below. *C. diphtheriae* C7(β) genomic DNA was amplified by PCR with forward primer *cat*-F12 (5′-TCATGAACTCTAGCACTC-3′) and reverse primer *cat*-R9 (5′-CCTATCAAATGTGAATC-3′) to produce fragment (a), with forward primer *cat*-F13 (5′-CAAAGCGGCGCGATCGCC-3′) and *cat*-R10 (5′-CAACTAAAGGTGGAGTGCT-3′) to produce fragment (b), and with forward primer *cat*-F14 (5′-TAAGCTATTAATCGATTC-3′) and reverse primer *cat*-R11 (5′-TGAGGAGCTGCTTCGGTGCG-3′) to produce fragment (c). Fragments (a), (b), and (c) were cloned into in the pCR2.1 TOPO vector (Invitrogen, Carlsbad, CA) to generate plasmids pT-*cat* A, pT-*cat* B, and pT-*cat* C, respectively, and the sequences of the cloned fragments (a), (b), and (c) were determined and shown to be identical with the corresponding regions of the genome sequence from *C. diphtheriae* NCTC13129. The *Eco*RI fragments corresponding to probes (a), (b), and (c) were excised from pT-*cat* A, pT-*cat* B, and pT-*cat* C, purified, and labeled with [γ-^32^P]ATP using T4 polynucleotide kinase (Promega, Madison, WI). The DNA probes (approximately 10^4^ cpm) were incubated at RT with varying amounts of purified OxyR protein from *C. diphtheriae* or *E. coli* in the binding reaction buffer reported previously [36]. When noted, 200 mM of dithiothreitol (DTT) was added to the reaction mixtures to reduce any disulfide bonds between cysteine residues in the OxyR protein. After incubation for 20-min, the reaction mixtures were analyzed using 5% non-denaturing polyacrylamide gels as described previously [36].

### DNaseI footprinting assay

A 244-bp DNA fragment was amplified from *C. diphtheriae* C7(β) genomic DNA by PCR using the *cat*-F13 forward primer described previously and the *cat*-R12 reverse primer (5′-CTTGTTCAGGATTGGATCGAC-3′) which had been 5′-labeled with ^32^P by prior treatment with [γ-^32^P]ATP and T4 polynucleotide kinase (Promega, Madison, WI). The resulting ^32^P-labeled DNA probe (approximately 10^6^ cpm, final concentration 0.12 nM) was incubated at RT with varying amounts of purified *C. diphtheriae* OxyR either treated with 200 mM DTT (reduced form of OxyR) or un-treated (air oxidized form of OxyR) in the binding reaction mixture described previously [37]. After incubation for 20-min, each reaction mixture was digested with 0.5 U of DNaseI (Promega, Madison, WI) for 2 min at 37°C, extracted with phenol/chloroform, and precipitated with ethanol. Samples of the DNaseI treated DNA were analyzed on an 8.3 M urea-6% polyacrylamide sequencing gel [36]. The nucleotide sequence was determined in a parallel reaction by the dideoxy-termination method using as template the pT-*cat* foot plasmid which contains the cloned 244-bp fragment used for footprinting, the 5′-^32^P-labeled *cat*-R12 reverse primer described above, and the Thermo Sequenase™ cycle sequencing kit (Amersham pharmacia biotech, Cleveland, OH).

### Western immunoblot assay

*E.coli* GC4468 or JL102 strains (harboring plasmids as indicated in the text) were grown in LB medium at 37°C and harvested during the exponential phase of growth (A600 between 0.7 and 0.9). *C. diphtheriae* C7(β) or C7(β) Δ*oxyR* strains (harboring plasmids as indicated in the text) were grown in PGT medium with 10 µM FeCl_3_ and harvested during the exponential phase of growth (A600 between 1.0 and 1.5). Cells were disrupted using by an ultrasonic liquid process (*E.coli*) or a bead-beater (*C. diphtheriae*), and electrophoresis (SDS-PAGE, 12% polyacrylamide) and electro-blotting of proteins were performed as described previously [26]. Each blot was treated with a 1/2000 dilution of polyclonal rabbit antibody against *E.coli* OxyR (kindly provided by Dr. Giesela Storz) followed by a 1/10,000 dilution of goat anti-rabbit IgG (Pierce, Rockford, IL), conjugated with horseradish peroxidase (HRP), and then visualized using a SuperSignal West Dura chemiluminescent substrate detection system supplied by Pierce. Protein concentrations of extracts were determined by NanoDrop spectrophotometer (Thermo Scientific, Wilmington, DE).

## Results

### The role of catalase in resistance to H_2_O_2_–induced oxidative stress in *C. diphtheriae*

**Figure 1:**
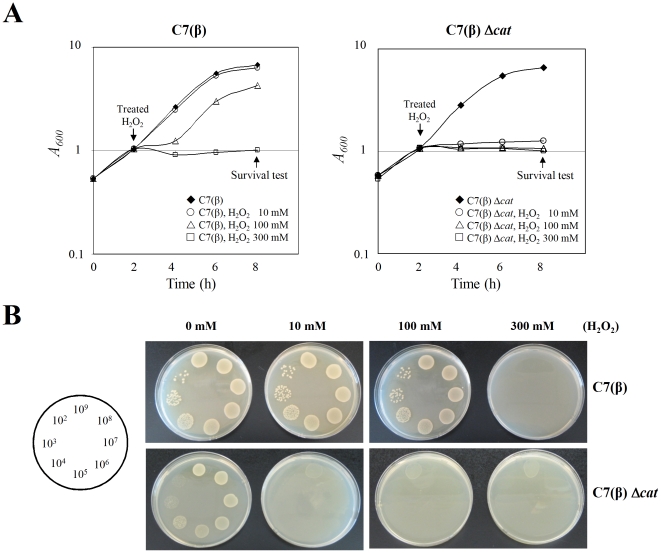
Increased susceptibility of the *C. diphtheriae* C7(β) Δ*cat* mutant to growth inhibition and killing by H_2_O_2_. A: Growth (A600) of wild-type *C. diphtheriae* C7(β) (left graph) and the isogenic C7(β) Δ*cat* mutant (right graph) in high-iron PGT medium. At the time points indicated by the downward arrows, H_2_O_2_ was added at 10 mM (○), 100 mM H_2_O_2_ (▵), or 300 mM H_2_O_2_ (□) or was omitted from controls (⧫). B: At the 8 h time points marked by upward arrows in (A) and (B), viability of wild-type *C. diphtheriae* C7(β) (top row) and C7(β) Δ*cat* (bottom row) was determined by spotting 30 µl samples from serial dilutions of each culture onto HIA as shown. Spotting cell number illustrate on the left. The photographs show bacterial growth after 14 h at 37°C.

*C. diphtheriae* is a catalase-positive gram-positive bacterium. As a baseline for studying the role of catalase in resistance of *C. diphtheriae* to H_2_O_2_–induced oxidative stress, we determined the effects of H_2_O_2_ on growth (Fig. 1A) and viability (Fig. 1B) of wild type *C. diphtheria*e C7(β). For bacteria growing in PGT medium with 10 µM FeCl_3_, adding H_2_O_2_ at a final concentration of 10 mM during the exponential phase of growth did not affect either growth rate or bacterial viability after 6 hours of exposure. Increasing the H_2_O_2_ concentration to 100 mM caused a lag of approximately 2 hours before resumption of normal growth but had little effect on viability after 6 hours of exposure. In contrast, when H_2_O_2_ was added at 300 mM, the wild-type cells showed complete growth arrest and no viable cells were present after 6 hours exposure. We observed similar effects of H_2_O_2_ on growth and viability of wild type *C. diphtheria*e C7(β) when the experiments were repeated in low-iron PGT medium or in HIBTW medium (data not shown). These results show that wild type *C. diphtheria*e C7(β) exhibits substantial tolerance to H_2_O_2_, presumably by degrading H_2_O_2_ to non-toxic products.

**Figure 2:**
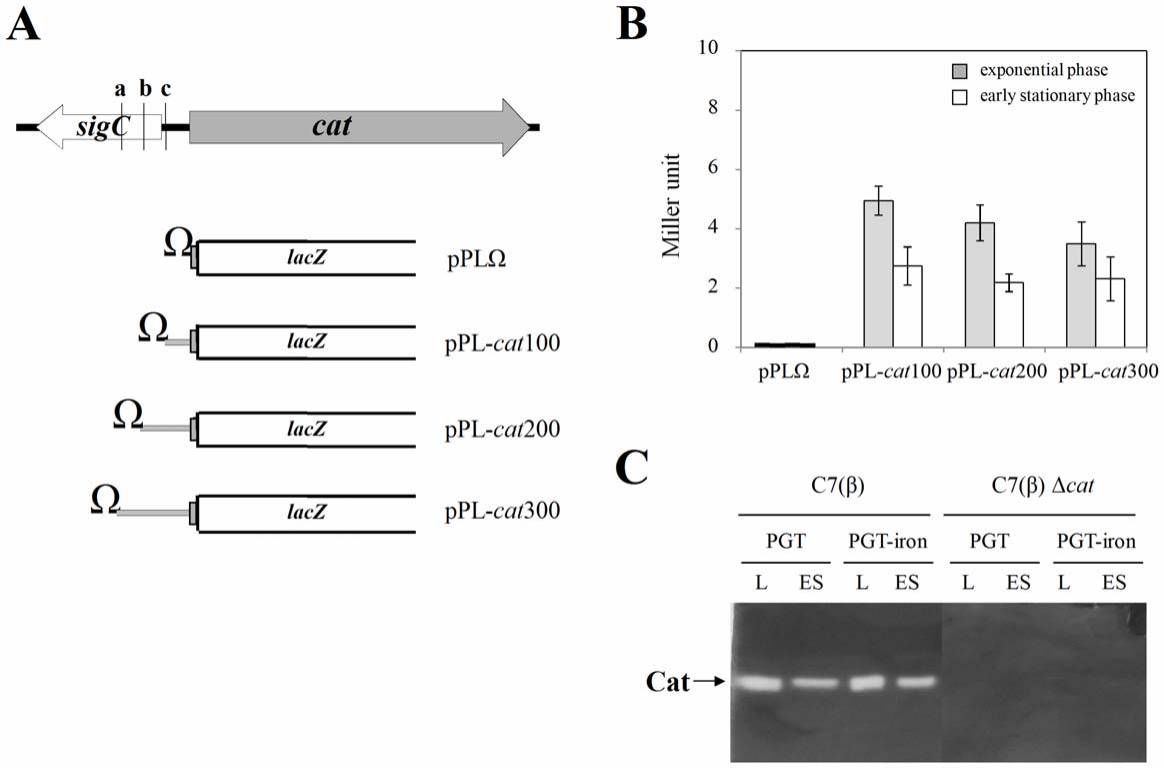
Structure of *cat*::*lacZ* transcriptional fusions and assessment of *cat* promoter activity and catalase activity in *C. diphtheriae* C7(β). A: Organization of the *sigC-cat* locus and schematic representation of *cat*::*lacZ* reporter plasmids. B: Histogram comparing β-galactosidase reporter activities for wild-type *C. diphtheriae* C7(β) carrying either the pPLΩ control plasmid, or the pPL-*cat*100, pPL-*cat*200 or pPL-*cat*300 reporter plasmid. Cultures grown in high-iron PGT medium were harvested either during exponential phase growth (shaded bars) or at early stationary phase (open bars). Error bars show standard deviations for β-galactosidase activities. C: Cultures of wild-type *C. diphtheriae* C7(β) or the C7(β) Δ*cat* mutant were grown to exponential phase (labeled L) or early stationary phase (labeled ES) in low-iron PGT (labeled PGT) or high-iron PGT (labeled PGT-iron). Samples were analyzed by native PAGE followed by in gel assays for catalase activity, which appears as the single bright band in each lane containing lysate from wild type C7(β).

The most obvious candidate enzymes for detoxifying H_2_O_2_ are catalases and peroxidases. A search of the published *C. diphtheriae* NCTC13129 genome sequence for homologs of catalase identified a single gene annotated as *cat* (DIP0281) (http://www.ncbi.nlm.nih.gov/gene/2649075). The *cat* gene ORF is separated by a 127-bp intervening sequence from the divergently oriented *sigC* ORF, which encodes an RNA polymerase sigma factor presumed to be involved in responses to environmental stress (Fig. 2A). We constructed an in-frame deletion in the *cat* gene of *C. diphtheriae* C7(β) by allelic exchange, and we used this Δ*cat* mutant strain to examine the role of catalase in the resistance *C. diphtheriae* to H_2_O_2_. In contrast with the results described previously with wild type C7(β), addition of H_2_O_2_ at 10 mM, 100 mM or 300 mM to exponential phase cultures of the C7(β) Δ*cat* mutant resulted in immediate cessation of growth and complete loss of viability after 6 hours of exposure (Figs. 1A and 1B). When methyl viologen (MV, a superoxide radical generating agent) was spotted on agar plates inoculated with sufficient numbers of wild type *C. diphtheriae* C7(β) or the C7(β) Δ*cat* mutant to produce confluent lawns during subsequent incubation, no significant differences were seen in the diameters of the zones of inhibition that developed (data not shown). These results indicate that catalase confers resistance to H_2_O_2_-induced stress, but not to MV-induced stress, in *C. diphtheriae*.

### Effects of growth phase and H_2_O_2_ on activity of the *cat* promoter and catalase

As an initial method to localize the *cat* promoter and measure its activity, we constructed and characterized a set of *cat::lacZ* transcriptional fusion reporter plasmids (Fig. 2A). Plasmids pPL-*cat*100, pPL-*cat*200, and pPL-*cat*300 contain, respectively, the 100-bp segment of the *sigC-cat* intergenic sequence immediately upstream from the *cat* ORF and the longer 200-bp and 300-bp DNA sequences which extend further upstream from the *cat* ORF. For *C. diphtheriae* C7(β) strains harboring either pPL-*cat*100, pPL-*cat*200, or pPL-*cat*300, the β-galactosidase reporter activity was significantly greater (approximately 1.8-fold) during the exponential phase of growth than during early stationary phase (P<.05) (Fig. 2B). The control plasmid pPLΩ, which does not contain any of the *sigC-cat* intergenic sequence, showed no β-galactosidase reporter activity. These data show that the functional *cat* promoter is located within the 100-bp segment of the *sigC-cat* intergenic sequence immediately upstream from the *cat* ORF and that the *cat* promoter is slightly but significantly more active during exponential growth than in stationary phase.

We measured catalase in exponential phase and early stationary phase cultures of wild type *C. diphtheriae* C7(β) by preparing extracts, subjecting them to electrophoresis on non-denaturing polyacrylamide gels, and performing in gel assays for catalase activity (Fig. 2C). The catalase from wild-type C7(β) migrated as a single band on the non-denaturing polyacrylamide gels and appeared, from the relative intensity of the reaction signals, to have ∼2–3 fold greater activity in the bacteria harvested during exponential phase compared to the bacteria harvested in the early stationary phase. Additional assays showed that catalase from *C. diphtheriae* does not have peroxidase activity and that catalase activity did not differ significantly in bacteria grown under high-iron vs. low-iron conditions (data not shown). Parallel controls performed with extracts from the C7(β) Δ*cat* mutant failed to demonstrate any catalase activity. These results indicate that the *cat* gene encodes the only catalase in *C. diphtheriae* C7(β) and that catalase is most likely regulated at the transcriptional level in *C. diphtheriae* C7(β).

**Figure 3:**
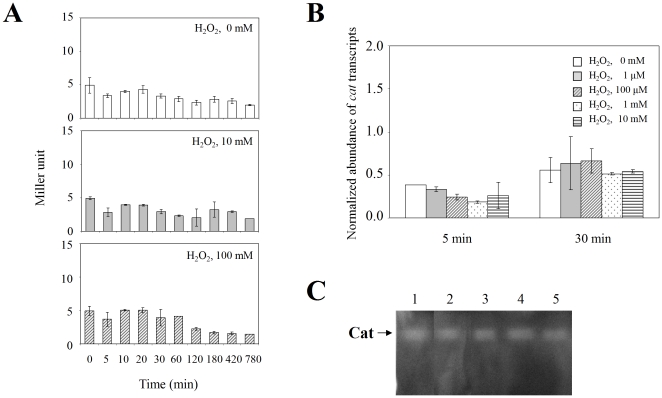
Exposure of wild-type *C. diphtheriae* C7(β) to H_2_O_2_ had little effect on *cat* promoter activity or catalase activity. A: Replicate cultures of *C. diphtheriae* C7(β) harboring pPL-*cat*200 were grown in high-iron PGT medium to exponential phase, and β-galactosidase activities were measured subsequently at the indicated times in a control without H_2_O_2_ (top histogram, open bars), and after addition of 10 mM H_2_O_2_ (middle histogram, shaded bars) or 100 mM H_2_O_2_ (bottom histogram, hatched bars). The error bars indicate standard deviations. B: Relative abundance of *cat* transcripts was determined with RNA extracted from mid-exponential phase cultures of wild type *C. diphtheriae* C7(β) after no treatment (open bars) or treatment for 5 min or 30 min with 1 µM H_2_O_2_ (shaded bars), 100 µM H_2_O_2_ (hatched bars), 1 mM H_2_O_2_ (dotted bars) or 10 mM H_2_O_2_ (lined bars). C: In gel assays for catalase were performed with extracts from mid-exponential phase cultures of wild type *C. diphtheriae* C7(β) after no treatment (lane 1) or treatment for 30 min with 1 µM H_2_O_2_ (lane 2), 100 µM H_2_O_2_ (lane 3), 1 mM H_2_O_2_ (lane 4) or 10 mM H_2_O_2_ (lane 5). Standard deviations were calculated from results of assays performed in triplicate.

As a first step in investigating whether the *cat* promoter in *C. diphtheriae* is activated in response to H_2_O_2_, we grew replicate cultures of wild-type C7(β) harboring pPL-*cat*200 in PGT medium with 10 µM FeCl_3_, and we compared β-galactosidase activity in individual cultures at various times after adding 10 mM or 100 mM H_2_O_2_ vs. a control culture without added H_2_O_2_ (Fig. 3A). In the H_2_O_2_–treated cultures, activity of the β-galactosidase reporter for the *cat* promoter showed a trend toward slight and progressive decrease over time, but none of the differences between the treated cultures and the untreated control cultures was statistically significant (P>0.05). To confirm and extend these findings, we used qRT-PCR to measure directly the relative abundance of *cat* transcripts in untreated control cultures and in cultures exposed for 5 or 30 min to H_2_O_2_ at concentrations of 1 µM, 100 µM, 1 mM and 10 mM (Fig. 3B). After 5 minutes, the relative abundance of *cat* transcripts decreased slightly in all of the H_2_O_2_–treated cultures, but the differences in *cat* transcript abundance between the treated and untreated cultures were statistically significant only for the 100 µM and 1 mM H_2_O_2_ treatment groups (p<0.05). After 30 min, the relative abundance of *cat* transcripts in treated cultures at each concentration of H_2_O_2_ did not differ significantly from the untreated controls (p>0.5).

In gel assays for catalase activity were performed on extracts prepared from control cultures of wild-type *C. diphtheriae* C7(β) during the exponential growth phase and from replicate cultures exposed for 30 minutes to H_2_O_2_ at 1 µM, 100 µM, 1 mM or 10 mM (Fig. 3C). By image analysis, catalase activity in the cultures treated with 1 µM, 100 µM, 1 mM and 10 mM H_2_O_2_ was estimated to be 87%, 85%, 92% and 96% of the catalase activity in the untreated control cultures, respectively. Extending the duration of H_2_O_2_ exposure to 2 h also failed to induce catalase activity (data not shown). In summary, treating *C. diphtheriae* C7(β) with H_2_O_2_ at concentrations from 1 µM to 10 mM resulted in insignificant to small but significant decreases in *cat* promoter activity and slight decreases in catalase activity. These findings differed dramatically from published results with *E coli* showing that exposure to H_2_O_2_ at concentrations ranging from 100 µM to 1 mM activates transcription of *katG* and production of catalase [38]. Our results provide no evidence that *cat* transcription or catalase production in *C. diphtheriae* is activated by exposure to H_2_O_2_.

### The role of OxyR in regulating the *cat* promoter and catalase in *C. diphtheriae*

Examination of the published genome sequence for *C. diphtheriae* NCTC13129 [39] identified one ORF (DIP1421) that encodes a putative transcriptional regulator similar to the H_2_O_2_-inducible OxyR from *Erwinia carotovora*. The predicted amino acid sequence of the *C. diphtheria*e OxyR encoded by DIP1421 is 100% identical with the putative *C. glutamicum* OxyR, 40% identical and 62% similar with *Streptomyces coelicolor* OxyR, 33% identical and 62% similar with *E.coli* OxyR, and 27% identical and 47% similar with *N. gonorrhoeae* OxyR (Fig. S1A). Each of these proteins has a highly conserved DNA binding domain characteristic of the LysR family of regulatory proteins as well as two conserved cysteine residues (Cys206 and Cys215 in *C. diphtheriae* OxyR) homologous to the Cys199 and Cys208 residues in *E.coli* OxyR that participate in redox sensing. No gene encoding a homolog of the *E. coli* OxyS protein is present in the *C. diphtheria*e genome. The *oxyR* gene in *C. diphtheriae* is flanked on the upstream side by the divergently transcribed *dirA* gene (which encodes an iron-repressible polypeptide) and on the downstream side by the convergently transcribed DIP1422 gene (which encodes a putative membrane protein) (Fig. S1B).

**Figure 4:**
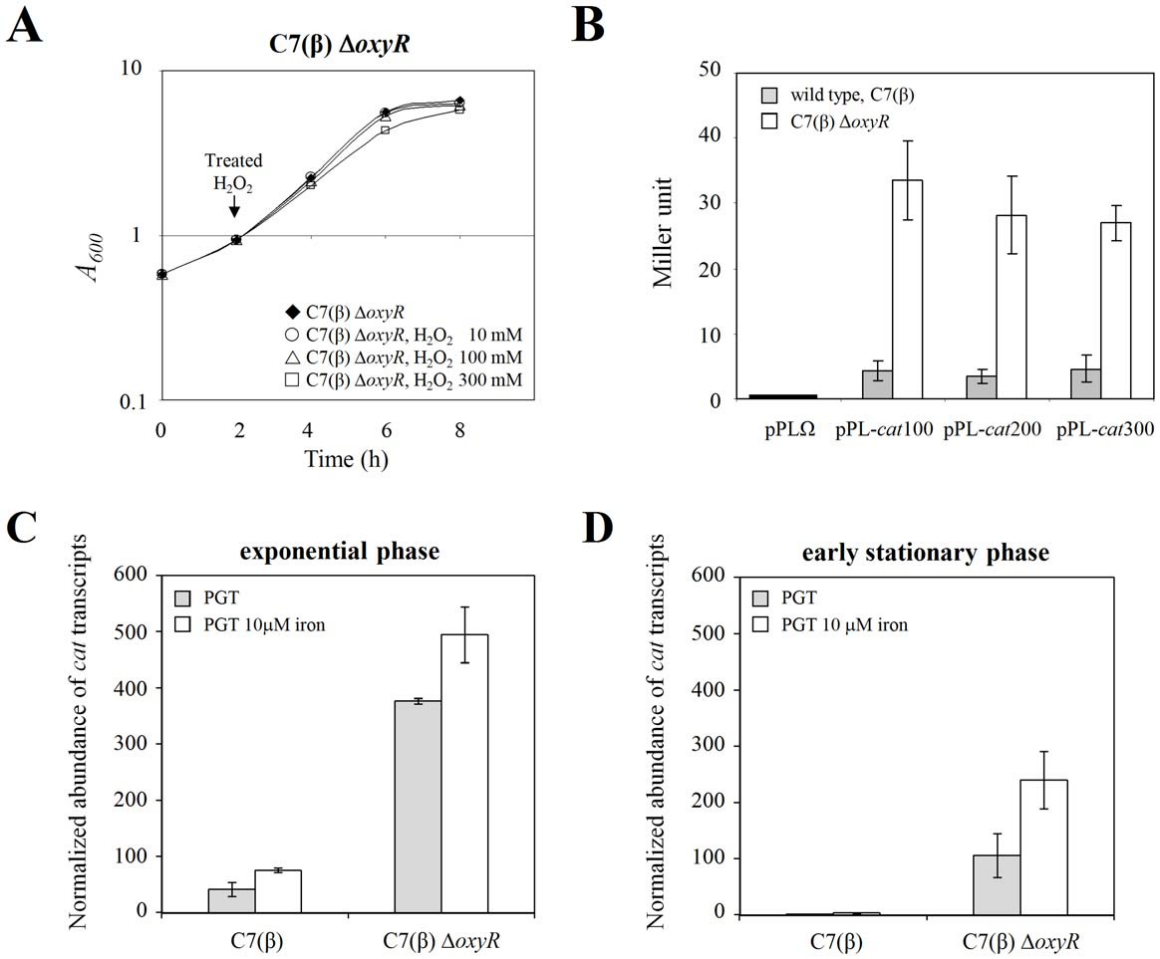
The *C. diphtheriae* C7(β) Δ*oxyR* mutant showed increased resistance to H_2_O_2_, increased activity of *cat* promoter reporters, and increased *cat* transcript abundance. A: Growth of the C7(β) Δ*oxyR* in high-iron PGT medium without (⧫) and with 10 mM H_2_O_2_ (○), 100 mM H_2_O_2_ (▵), or 300 mM H_2_O_2_ (□) added during exponential growth (downward arrow). B: β-galactosidase activities during exponential phase growth in high-iron PGT medium of wild-type *C. diphtheriae* C7(β) (shaded bars) and C7(β) Δ*oxyR* (open bars) harboring plasmid pPLΩ, pPL-*cat*100, pPL-*cat*200 or pPL-*cat*300. Error bars show standard deviations of the mean (n = 3). C and D: Normalized abundance of *cat* transcripts in wild-type *C. diphtheriae* C7(β) and the C7(β) Δ*oxyR* mutant during exponential phase growth (C) or early stationary phase growth (D) in low-iron PGT medium (shaded bars) or high-iron PGT medium (open bars). Error bars show standard deviations of the mean (n = 3).

We constructed an in-frame deletion mutation in the *oxyR* gene of *C. diphtheriae* C7(β) by allelic exchange (Fig. S1B). We examined susceptibility of the C7(β) Δ*oxyR* mutant to oxidative stress by comparing the effects on growth of H_2_O_2_ at final concentrations of 10 mM, 100 mM, and 300 mM in replicate exponential phase cultures vs. control cultures without exposure to H_2_O_2_. In striking contrast with the findings for wild type C7(β) shown previously (Fig. 1A), the C7(β) Δ*oxyR* mutant grew as well after exposure to 10 mM or 100 mM H_2_O_2_ as it did without exposure to H_2_O_2_, and it showed only a slight decrease in growth after exposure to 300 mM H_2_O_2_ (Fig. 4A). The tolerance of the C7(β) Δ*oxyR* mutant to H_2_O_2_ was comparable in low-iron PGT medium, PGT medium with 10 µM FeCl_3_, and HIBTW medium (data not shown). Introducing a cloned wild type *oxyR* allele into the C7(β) Δ*oxyR* mutant by integrating the pK-PIM-*oxyR*_C7(β)_ plasmid into a chromosomal *attB* site [30] complemented the Δ*oxyR* mutation and restored the growth phenotype after exposure to H_2_O_2_ to wild type (data not shown).

We used two different methods to demonstrate the effects of OxyR on activity of the *cat* promoter. First, we introduced the pPL-*cat*100, pPL-*cat*200, or pPL-*cat*300 reporter plasmid (or the pPLΩ control plasmid) into wild type *C. diphtheriae* C7(β) and the C7(β) Δ*oxyR* mutant and measured β-galactosidase activity as a surrogate for *cat* promoter activity during exponential growth of each strain in PGT medium with 10 µM FeCl_3_ (Fig. 4B). Each of the C7(β) Δ*oxyR* derivatives exhibited a statistically significant 6–8 fold increase in β-galactosidase activity compared with the corresponding isogenic wild-type C7(β) derivative (P<0.05), and no β-galactosidase activity was present in C7(β) or C7(β) Δ*oxyR* harboring pPLΩ. To confirm and extend these findings, we used real-time quantitative RT-PCR assays to compare the relative abundance of *cat* transcripts in wild type C7(β) and the isogenic C7(β) Δ*oxyR* mutant during exponential growth (Fig. 4C) and early stationary phase (Fig. 4D), both in low-iron PGT medium and in PGT medium with 10 µM FeCl_3_. The normalized abundance of *cat* transcripts was significantly greater by 6–8 fold in C7(β) Δ*oxyR* than in C7(β) under each set of growth conditions (P<0.05). For each strain, the normalized abundance of *cat* transcripts declined by two-fold or more during transition from the exponential growth phase to early stationary phase, confirming our previous finding using reporter plasmids in wild type C7(β) (Fig. 2B). Furthermore, in C7(β) and C7(β) Δ*oxyR* during exponential and early stationary phase growth, the normalized abundance of *cat* transcripts exhibited a small (two-fold or less) but significant (P<0.05) increase under high-iron vs. low-iron conditions.

**Figure 5:**
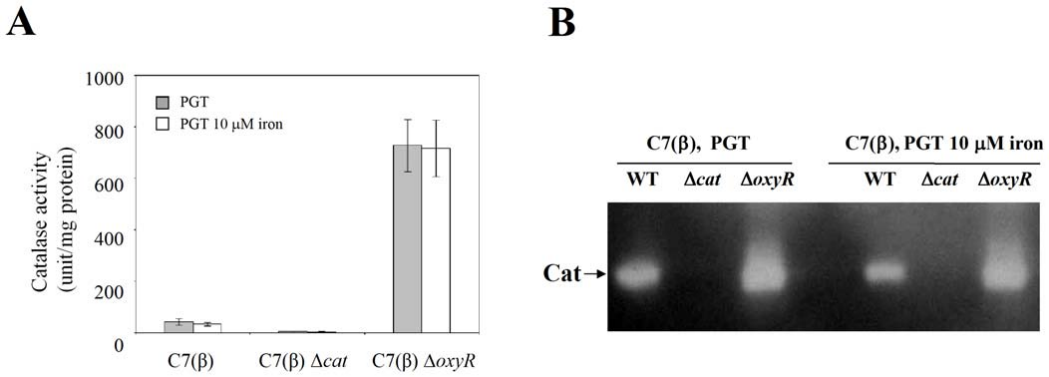
Assays for catalase activity. A: Wild-type *C. diphtheriae* C7(β), the C7(β) Δ*cat* mutant, and the C7(β) Δ*oxyR* mutant were grown in low-iron PGT medium (shaded bars) and high-iron PGT medium to exponential phase, and quantitative assays for catalase activity were performed in triplicate. Error bars show standard deviations of the mean. B: Samples from each bacterial extract were separated by non-denaturing PAGE, and in gel assays for catalase revealed a single intense band of activity in the extract from the C7(β) Δ*oxyR* mutant, a significantly weaker single band of activity from wild type C7(β), and no detectable activity from the C7(β) Δ*cat* mutant.

We performed direct biochemical assays for catalase activity with extracts prepared from exponential phase cultures of *C. diphtheriae* C7(β), C7(β) Δ*cat*, and C7(β) Δ*oxyR* grown in low-iron PGT medium and in PGT medium with 10 µM FeCl_3_ (Fig. 5A). No catalase activity was detected in C7(β) Δ*cat*, confirming that *cat* is the sole gene responsible for production of catalase in *C. diphtheriae* C7(β). The specific activity of catalase was approximately 15- to 20-fold greater in C7(β) Δ*oxyR* than in wild type C7(β), but growth in low-iron vs. high-iron medium had no significant effect on catalase activity (P>0.05). Analysis of these extracts by non-denaturing PAGE and in gel assays for catalase activity supported these conclusions (Fig. 5B). Taken together, these findings demonstrate that OxyR functions as a repressor of *cat* transcription, support the conclusion that regulation of catalase occurs primarily at the level of transcription in *C. diphtheriae*, and provide no evidence that OxyR functions as an activator of *cat* transcription in *C. diphtheriae*.

Both in wild type C7(β) and in C7(β) Δ*oxyR*, we found small but statistically significant (P<0.05) increases in the normalized abundance of *cat* transcripts under high iron vs. low-iron growth conditions (Figs. 4C and 4D) that were not accompanied by increases in catalase activity (Figs. 5A and 5B). These effects were independent of OxyR, since they occurred both in C7(β) and in C7(β) Δ*oxyR*.

We also showed by real-time quantitative RT-PCR assays that the relative abundance of *sigC* transcripts was about 1.6-fold greater in C7(β) Δ*oxyR* than in C7(β) during exponential phase growth in low-iron PGT medium, and about 2-fold greater in C7(β) Δ*oxyR* than in C7(β) during exponential phase growth in high-iron PGT medium (data not shown). These modest differences suggest that OxyR has little role in regulating transcription of *sigC*, notwithstanding the fact that *cat* and *sigC* are separated only by a short intergenic region and are divergently transcribed (Fig. 2A).

### Molecular analysis of OxyR interaction with the *cat* promoter in *C. diphtheriae*

**Figure 6:**
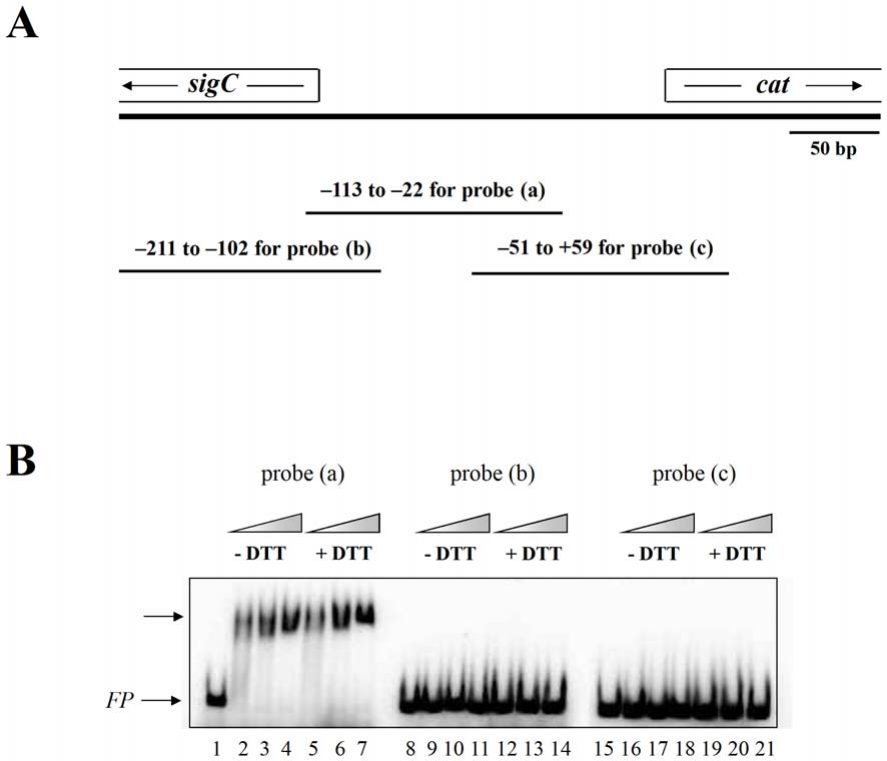
Assessment of binding of *C. diphtheriae* OxyR to segments of the *cat* regulatory region by gel mobility shift assays. A: Map of the *sigC*-*cat* locus showing the locations of probes (a), (b) and (c) and nucleotide positions of their 5′ and 3′ ends relative to the +1 nucleotide in the *cat* ORF. B: Gel mobility shift assays were performed with the indicated probes (at 21 pM) either without OxyR (lanes 1, 8, and 15) or with OxyR at 56 pM (lanes 2, 5, 9, 12, 16, and 19), 140 pM (lanes 3, 6, 10, 13, 17, and 20), or 280 pM (lanes 4, 7, 11, 14, 18, and 21). All OxyR concentrations are monomer equivalents. The triangles show the increasing concentrations of OxyR. DTT was present at 200 mM in lanes 5–7, 12–14, and 19–21, and DTT was absent from the other lanes. The lower arrow labeled (*FP*) indicates the free probes, and the top arrow shows the complex of probe (a) with air-oxidized OxyR (lanes 2–4, −DTT) or reduced OxyR (lanes 5–7, +DTT).

Our previous experiments using pPL-*cat*100, pPL-*cat*200, and pPL-*cat*300 as reporters for *cat* promoter activity (Figs. 2B and Fig. 4B) showed that the *cat* promoter is located within the 100 bp segment of the *sigC*-*cat* intergenic region that lies immediately upstream from the *cat* ORF start codon. In an effort to predict a possible OxyR binding target by bio-informatic methods, we used the MEME program (http://meme.sdsc.edu./meme/) to search the *sigC*-*cat* intergenic region for homologs of the known 50 bp *E.coli* OxyR binding target (5′-CAATAATAAGCAAATCCTTTAATTGTACAATTATTGTTAAGAATTACCTA-3′) [40]. The best match, extending from 52–103 bp upstream from the *cat* ORF start codon, was 75% similar to the *E.coli* OxyR binding target. As a first step toward testing whether *C. diphtheriae* OxyR can bind to this predicted site, we designed three overlapping 110 bp DNA probes for use in gel mobility shift assays. Probe (a), extending from nucleotide −113 to −22 with respect to the +1 nucleotide of the *cat* ORF start codon, was centered on the predicted binding site described above (Fig. 6A). Probe (b), extending from nucleotide −211 to −102, and probe (c), extending from nucleotide −51 to +59, lie immediately upstream and immediately downstream, respectively, from the predicted binding site described above (Fig. 6A). Gel mobility shift assays showed that purified *C. diphtheriae* OxyR bound to and shifted probe (a) but not probe (b) or probe (c) (Fig. 6B). The position of the shifted probe (a) did not change as the concentration of *C. diphtheriae* OxyR (calculated as monomer) added to the gel shift reaction mixture increased from 56 pM to 280 pM, suggesting that OxyR formed complexes with DNA of uniform stoichiometry within this range of experimental conditions. Purified *C. diphtheriae* OxyR shifted probe (a) to the same extent and to the same position whether or not 200 mM DTT was present in the gel shift reaction mixture. These experiments were performed on three different occasions, with the same results. These findings suggest that purified *C. diphtheriae* OxyR can bind to the *cat* promoter region either in its reduced or oxidized form.

**Figure 7:**
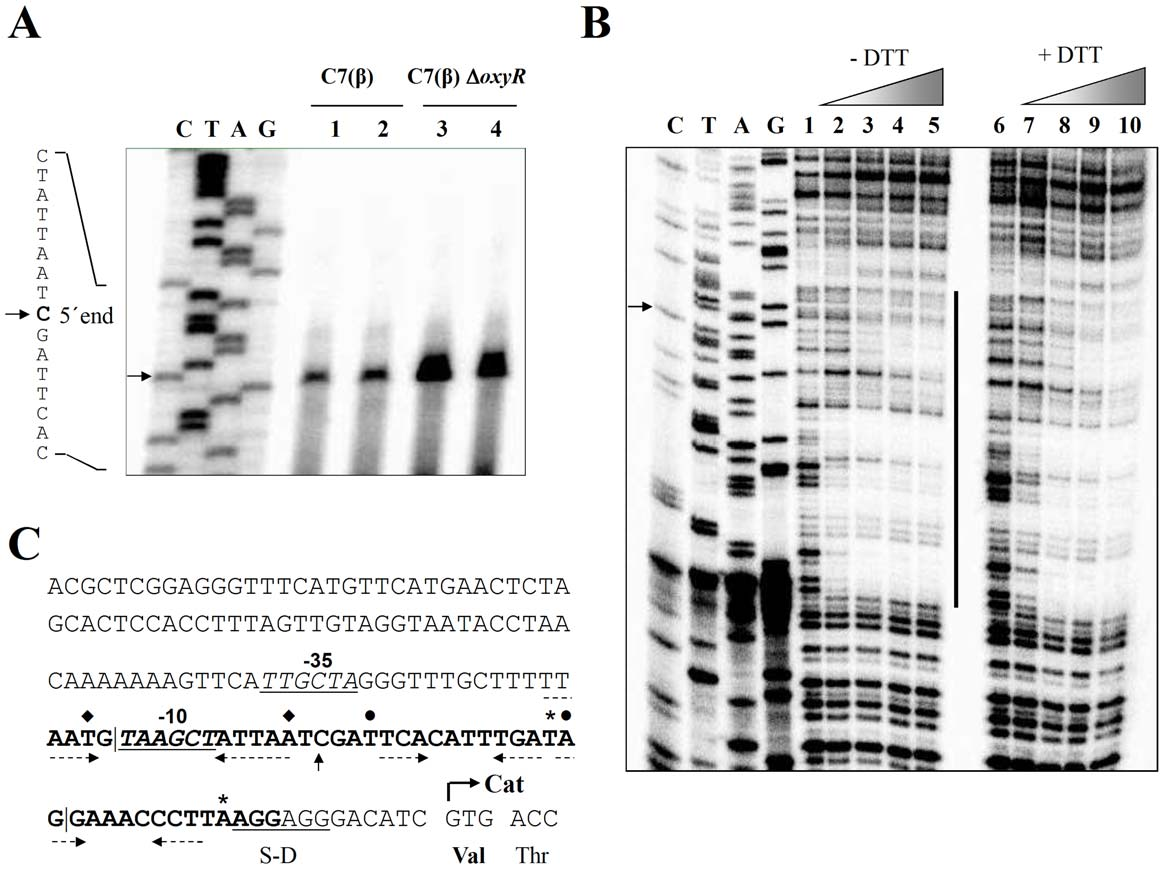
Characterization of the *oxyR* promoter-operator region. A: The 5′ end of the *cat* transcript was mapped by primer extension assays in wild-type *C. diphtheriae* C7(β) (lanes 1 and 2) and C7(β) Δ*oxyR* (lanes 3 and 4) grown to exponential phase in low-iron PGT medium (lanes 1 and 3) and high-iron PGT medium (lanes 2 and 4). The same ^32^P-labeled reverse primer was used for the sequence ladder (labels C, T, A, and G correspond to nucleotides in the sense strand of the *cat* gene) and the primer extension products (lanes 1, 2, 3 and 4). In the partial sequence of the sense strand shown on the left, the arrow marks the C residue (bold font) at the 5′ end of the *cat* transcript. B: DNaseI footprint of the OxyR binding site in the *cat* regulatory region. Controls in lanes 1 and 6 contained no OxyR. Triangles at the top show increasing concentrations of OxyR (calculated as the monomer), which were 0.28 nM for lanes 2 and 7, 0.56 nM for lanes 3 and 8, 1.4 nM for lanes 4 and 9, and 2.8 nM for lanes 5 and 10. Lanes 1–5 had no DTT (labeled −DTT), and lanes 6–10 had 200 mM DTT (labeled +DTT). The vertical line shows the sequence that was protected by OxyR from DNaseI cleavage. The sequence ladder is labeled as in (A), and the arrow marks the C residue corresponding to the 5′ end of the *cat* transcript. C: The DNA sequence includes the *cat* regulatory region and part of the *cat* ORF. The C residue at the 5′ end of the *cat* transcript is marked by an up arrow; the putative ribosome-binding site (labeled S–D) is underlined; the putative −10 promoter hexamer (labeled −10) is italicized, bold and underlined; the putative −35 promoter hexamer (labeled −35) is italicized and underlined; and the 46-bp OxyR footprint (−55 to −10 relative to the start of the *cat* ORF) is bold. The upstream and downstream vertical lines within the OxyR footprint show the locations of the 5′ end of probe (c) and the 3′ end of probe (a), respectively. Three possible T-N11-A target motifs for OxyR binding within the OxyR footprint sequence are marked by filled diamonds, filled circles, and asterisks, respectively, above their 5′ T and 3′ A residues. The horizontal dashed arrows show short palindromic sequences associated with these possible T-N11-A motifs. The translational start for the *cat* ORF is shown by a bent arrow.

Next, we mapped the 5′ end of the *cat* transcripts in total RNA isolated from exponential phase cultures of wild-type *C. diphtheriae* C7(β) and the C7(β) Δ*oxyR* mutant grown in low-iron PGT medium and in PGT medium with 10 µM FeCl_3_ (Fig. 7A). In both bacterial strains and in both growth media, the 5′ end of the *cat* transcript was the C residue at position −39 relative to the +1 position in the *cat* ORF start codon. The intensity of the band corresponding to the primer extension product was greater with RNA from C7(β) Δ*oxyR* than from wild type C7(β), reflecting the greater abundance of *cat* transcripts in C7(β) Δ*oxyR* (Fig. 4C).

To define more precisely the binding target for *C. diphtheriae* OxyR in the *cat* promoter region, we performed DNaseI footprinting assays. Either in the presence or absence of 200 mM DTT, binding of OxyR protected a 46-bp AT-rich sequence extending from position −55 to −10 with respect to the +1 position in the *cat* ORF start codon (Fig. 7B). The extent of protection increased progressively as the concentration of OxyR in the reaction mixture increased from 0.28 nM to 2.8 nM. The OxyR binding site demonstrated by the DNaseI protection assays was immediately downstream from, but did not overlap with, the putative binding site predicted by the bio-informatics analysis described above based on homology with the binding site for *E. coli* OxyR.

The 46 bp sequence corresponding to the DNaseI footprint is shown in bold font in Fig. 7C. Both the G residue at position −22 that corresponds to the 3′ end of probe (a) and the T residue at position −51 that corresponds to the 5′ end of probe (c) lie within this DNaseI footprint. The upstream and downstream vertical lines inserted into the sequence in Fig. 7C show the location of the 5′ end of probe (c) and the 3′ end of probe (a), respectively. The ability of *C. diphtheriae* OxyR to shift the mobility of probe (a) (Fig. 6B) indicates that the 13 nucleotides at the 3′ end of the DNaseI footprint sequence are not required for OxyR binding to the *cat* promoter region, although they might contribute to optimal binding. Conversely, the failure of *C. diphtheriae* OxyR to shift the mobility of probe (c) (Fig. 6B) suggests that some or all of the 4 nucleotides at the 5′ end of the DNaseI footprint sequence might be required for OxyR binding. To investigate this possibility further, we used PCR to prepare a nested set of seven DNA amplicons that shared the downstream end of probe (c) at position +59 with respect to the relative to the +1 position in the *cat* ORF start codon but differed at their upstream ends. The shortest amplicon was identical to probe (c), and the six longer amplicons extended 2 bp, 4 bp, 9 bp, 14 bp, 19 bp or 29 bp further upstream to terminate at positions −53, −55, −60, −65, −70 or −80, respectively. In gel mobility shift assays, *C. diphtheriae* OxyR failed to shift the mobility of the amplicon that was identical to probe (c) but did shift the mobility of all of the longer amplicons (data not shown). We conclude, therefore, that the sequence extending downstream from the T residue at position −53 in Fig. 7C contains the minimal essential binding site for *C. diphtheriae* OxyR.

### Comparison of Δ*cat* and Δ*oxyR* mutations in *C. diphtheriae* and *C. glutamicum*

**Figure 8:**
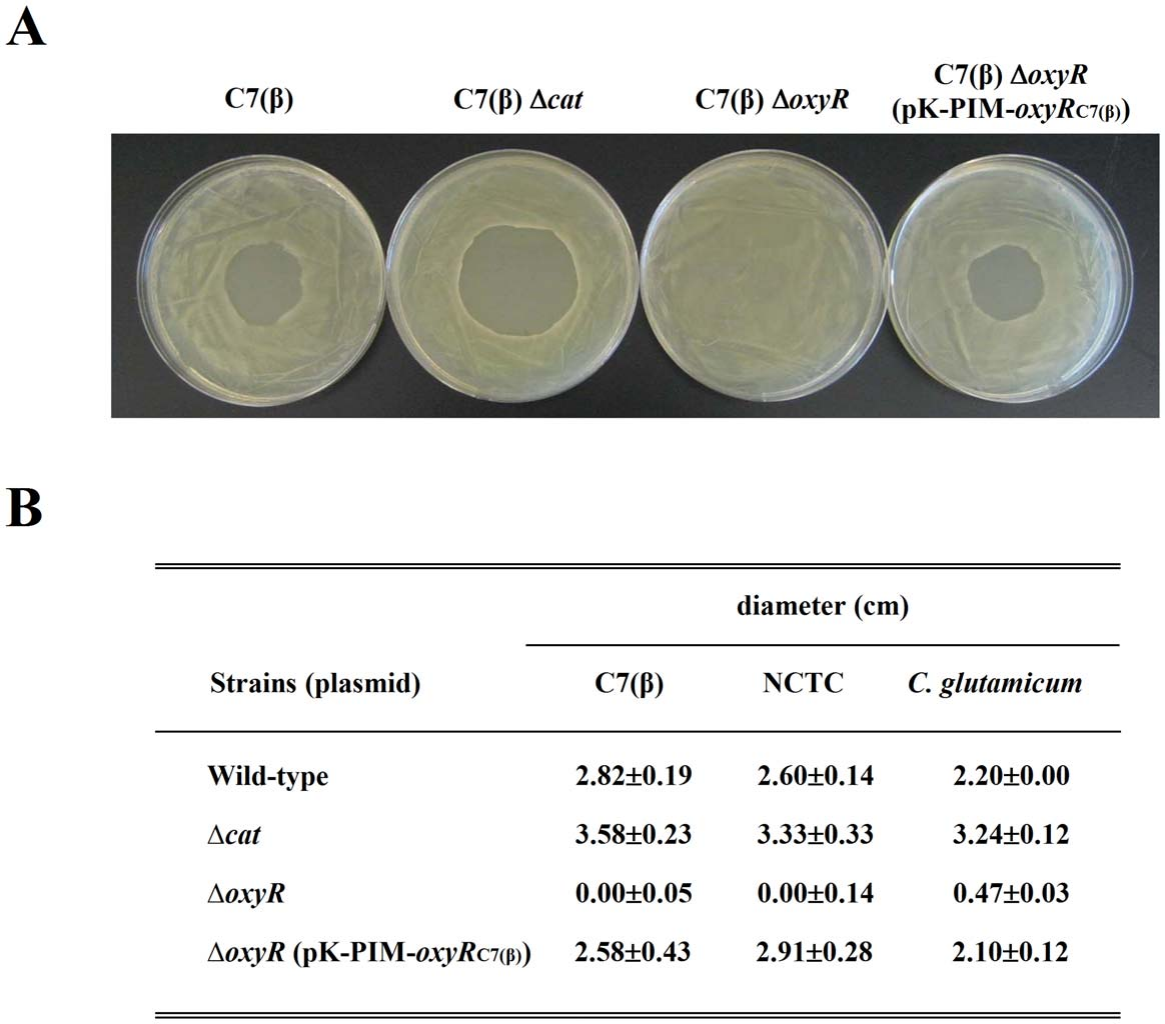
Susceptibility to H_2_O_2_ of wild type, Δ*cat*, Δ*oxyR* and complemented Δ*oxyR* variants of *C. diphtheriae* C7(β), *C. diphtheriae* NCTC13129, and *C. glutamicum* ATCC13032. A: Agar-diffusion growth-inhibition assays were performed in triplicate to test the tolerance of each of the indicated *C. diphtheriae* C7(β) variants to H_2_O_2_. Representative plates were photographed after overnight incubation at 37°C. Similar experiments were repeated with wild type and comparable isogenic derivatives of *C. diphtheriae* NCTC13129 and *C. glutamicum*, except that 4 M H_2_O_2_ instead of 1 M H_2_O_2_ was used for the tests with *C. glutamicum* (data not shown). B: The diameter of each zone of inhibition (in cm) was measured in three different directions. Mean diameters and standard deviations of the mean (n = 3) for each bacterial strain are presented in the table.

*C. diphtheriae* C7(β) has been maintained in laboratories since the 1950s [24] and has been used by many investigators as a reference strain for research. To determine whether our results with C7(β) are representative of other isolates of *C. diphtheriae* or of another *Corynebacterium* species, we performed confirmatory studies with the recent clinical isolate *C. diphtheriae* NCTC13129 (used for the first genome sequence of this species) and with *C. glutamicum* ATCC13120 (a nonpathogenic *Corynebacterium* widely used in biotechnology). For each of these reference strains, we constructed an isogenic Δ*cat* and an isogenic Δ*oxyR* in-frame single deletion mutant, and we complemented the Δ*oxyR* mutant by integration of pK-PIM-*oxyR*_C7(β)_ into a chromosomal *attB* site. We then compared the newly constructed wild type, mutant and complemented-mutant variants of *C. diphtheriae* NCTC13129 and *C. glutamicum* ATCC13120 with the homologous C7(β) variants described previously. For this purpose, we used agar-diffusion growth-inhibition assays to test the tolerance of each of these bacterial variants to H_2_O_2_. Fig. 8A shows photographs of representative plates from tests with wild type C7(β), with the isogenic Δ*cat* and Δ*oxyR* mutants, and with the Δ*oxyR* mutant complemented with pK-PIM-*oxyR*_C7(β)_. Fig. 8B presents the results from quantitative measurements of the diameters of the growth inhibition zones from tests performed with the wild type *C. diphtheriae* C7(β), *C. diphtheriae* NCTC13129 and *C. glutamicum* ATCC13120 strains and the mutant and complemented-mutant strains derived from them. In all cases, the Δ*cat* mutants exhibited increased susceptibility to H_2_O_2_, the Δ*oxyR* mutants showed dramatically decreased susceptibility to H_2_O_2_, and the susceptibility to H_2_O_2_ of the complemented Δ*oxyR* mutants was comparable to that of their wild type parental strains. Complementation tests with pK-PIM-*oxyR* plasmids carrying the wild type *oxyR* allele from *C. diphtheriae* NCTC13129 or *C. glutamicum* ATCC13120 gave equivalent results to those described above with the pK-PIM-*oxyR*_C7(β)_ plasmid (data not shown). These findings support the conclusions that, in both *C. diphtheriae* and *C. glutamicum*, catalase protects against H_2_O_2_–induced oxidative stress, *oxyR* functions as a repressor of *cat*, and *oxyR* clones from *C. diphtheriae* and *C. glutamicum* are expressed in a comparable manner.

### *C. diphtheriae* OxyR and *E.coli* OxyR are not functionally equivalent

**Figure 9:**
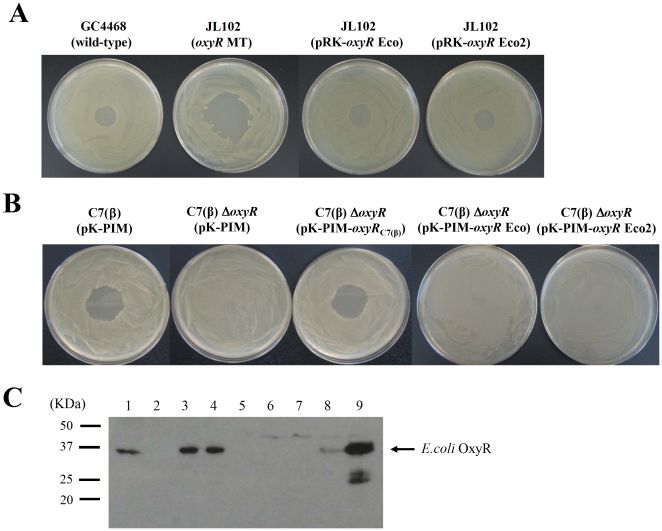
OxyR from *E. coli* does not complement the Δ*oxyR* mutation in *C. diphtheriae*. A: The phenotypes of wild type *E. coli* strain GC4468 and its isogenic Δ*oxyR* derivative JL102 in H_2_O_2_ agar-diffusion growth-inhibition assays are shown. The mutant phenotype of *E. coli* JL102 was complemented by the cloned *E. coli oxyR* gene expressed either from its native *E. coli oxyR* promoter (in pRK-*oxyR* Eco) or from the heterologous *C. diphtheriae oxyR* promoter (in pRK-*oxyR* Eco2). B: The phenotypes of wild type *C. diphtheriae* C7(β) containing pK-PIM and its isogenic Δ*oxyR* derivative containing the pK-PIM are shown. *C. diphtheriae* OxyR expressed from its native promoter in the pK-PIM-*oxyR*_C7(β)_ clone complemented the mutant phenotype of C7(β) Δ*oxyR*. In contrast, *E. coli* OxyR expressed either from its native promoter in pK-PIM-*oxyR* Eco or from the heterologous *C. diphtheriae oxyR* promoter in pK-PIM-*oxyR* Eco2 failed to complement the mutant phenotype of C7(β) Δ*oxyR*. C: SDS-PAGE and Western blot analysis with rabbit antiserum against *E.coli* OxyR was performed on extracts from exponential phase cultures of the following bacterial stains: *E.coli* GC4468 (lane 1), *E.coli* JL102 (lane 2), *E.coli* JL102 harboring pRK-*oxyR* Eco (lane 3), *E.coli* JL102 harboring pRK-*oxyR* Eco2 (lane 4), *C. diphtheriae* C7(β) containing pK-PIM (lane 5), *C. diphtheriae* C7(β) Δ*oxyR* containing pK-PIM (lane 6), *C. diphtheriae* C7(β) Δ*oxyR* containing pK-PIM-*oxyR*_C7(β)_ (lane 7), *C. diphtheriae* C7(β) Δ*oxyR* containing pK-PIM-*oxyR* Eco (lane 8), and *C. diphtheriae* C7(β) Δ*oxyR* containing pK-PIM-*oxyR* Eco2 (lane 9). Precision plus protein dual color standard (Bio-Rad) was used for molecular mass standards.

We used agar-diffusion growth-inhibition assays and complementation tests similar to those described above to evaluate the ability of the *E. coli oxyR* gene to substitute functionally for the *C. diphtheriae oxyR* gene. For tests in *E. coli* we used the wild type strain GC4468 and its isogenic *oxyR* mutant strain JL102 [41]. Preliminary control experiments on LB agar plates confirmed that the *oxyR* mutant JL102 exhibited greater susceptibility to growth inhibition by H_2_O_2_ than the parental strain GC4468 (Fig. 9A) as described previously [41]. For complementation studies in *E. coli*, we cloned the *E. coli oxyR* gene with its native promoter region from GC4468 genomic DNA into pRK415 (a broad-host range, low copy number plasmid [42]) to generate pRK-*oxyR* Eco. We also constructed pRK-*oxyR* Eco2, which fuses a 500-bp *oxyR* promoter fragment from *C. diphtheriae* C7(β) to the *oxyR* ORF from *E. coli* GC4468, thereby replacing the native *E. coli oxyR* promoter region with the heterologous *C. diphtheriae oxyR* promoter region. Introducing either pRK-*oxyR* Eco or pRK-*oxyR* Eco2 into *E. coli* JL102 complemented its *oxyR* defect and restored its H_2_O_2_ susceptibility phenotype to wild type (Fig. 9A). Therefore, the structural gene for *E. coli oxyR* is functional both in the pRK-*oxyR* Eco clone and pRK-*oxyR* Eco2 clone, and biologically active *E. coli* OxyR protein can be produced in *E. coli* when the *oxyR* structural gene is expressed either under control of its native promoter or the heterologous *oxyR* promoter from *C. diphheriae* C7(β).

Next, we subcloned the native and hybrid *oxyR* loci from pRK-*oxyR* Eco and pRK-*oxyR* Eco2 into pK-PIM to generate pK-PIM-*oxyR* Eco and pK-PIM-*oxyR* Eco2, respectively. When we introduced each of these plasmids into *C. diphtheriae* C7(β) Δ*oxyR*, neither was able to complement the Δ*oxyR* mutant phenotype, in contrast to the positive complementation result obtained with the pK-PIM-*oxyR*_C7(β)_ control (Fig. 9B). Finally, we performed Western blots with a rabbit antibody against *E. coli* OxyR to investigate the amounts of *E. coli* OxyR protein produced in the *E. coli* and *C. diphtheriae* strains described above (Fig. 9C). OxyR was present in wild type *E. coli* GC4468 but not in the isogenic *oxyR* mutant JL102. The amounts of OxyR in extracts from *E. coli* JL102 harboring pRK-*oxyR* Eco or pRK-*oxyR* Eco2 were slightly greater than in wild type GC4468. Antibody to the *E. coli* OxyR protein failed to detect *C. diphtheriae* OxyR in the Western blots with extracts from C7(β) or C7(β) Δ*oxyR*. *E. coli* OxyR was detected in extracts from *C. diphtheriae* C7(β) Δ*oxyR* harboring pK-PIM-*oxyR* Eco or pK-PIM-*oxyR* Eco2, and the amount of *E. coli* OxyR made in C7(β) Δ*oxyR* harboring pK-PIM-*oxyR* Eco2 was significantly greater that the amount detected in *E. coli* JL102 harboring pRK-*oxyR* Eco or pRK-*oxyR* Eco2. Taken together, these results demonstrated that *E. coli* OxyR encoded by a functional native or hybrid *oxyR* locus was produced in *C. diphtheriae* but failed to complement the Δ*oxyR* phenotype or substitute for *C. diphtheriae* OxyR as a repressor of transcription of the *cat* gene.

**Figure 10:**
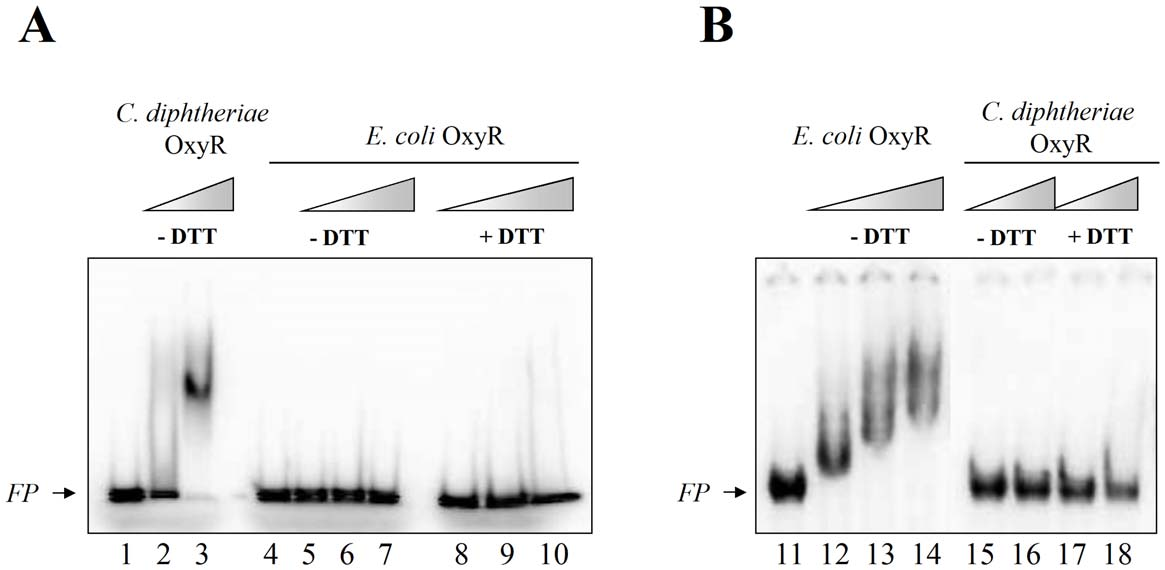
*C. diphtheriae* OxyR and *E. coli* OxyR exhibit specific binding to their cognate *cat* regulatory regions in gel mobility shift assays. A: Probe (a) containing the OxyR binding site (at 21 pM) exhibited a gel shift with *C. diphtheriae* OxyR but not with *E. coli* OxyR. Lanes 1 and 4 contained no OxyR. The positive controls in lanes 2–3 contained *C. diphtheriae* OxyR at 56 pM and 280 pM (calculated as monomer), respectively, without DTT. Lanes 5–7 contained *E. coli* OxyR at 58 pM, 145 pM and 290 pM (calculated as monomer), respectively, without DTT. Lanes 8–10 contained *E. coli* OxyR at 58 pM, 145 pM, and 290 pM, respectively, with 200 mM DTT. B: The 300-bp probe containing the *E.coli katG* regulatory region (at 21 pM) exhibited a gel shift with *E. coli* OxyR but not with *C. diphtheriae* OxyR. Lanes 11 and 15 contained no OxyR. The positive controls in lanes 12–14 contained *E. coli* OxyR at 58 pM, 145 pM and 290 pM, respectively, without DTT (positive control). Lanes 15–16 contained *C. diphtheriae* OxyR at 56 pM and 280 pM, respectively, without DTT. Lanes 17–18 contained *C. diphtheriae* OxyR at 56 pM and 280 pM, respectively, with 200 mM DTT. The arrows marked *FP* mark position of the free DNA probes.

Finally, we used gel mobility shift assays to test directly the ability of purified OxyR from *C. diphtheriae* and purified OxyR from *E. coli* to bind to target sequences in DNA probe (a) (Fig. 6A) from the *cat* promoter region of *C. diphtheriae* (Fig. 10A) and to a 300 bp DNA fragment from the *katG* regulatory region of *E. coli* (Fig. 10B). The purified OxyR from *C. diphtheriae* bound to and shifted the mobility of probe (a), in agreement with results shown previously in Fig. 6B, but OxyR from *E. coli* failed to shift probe (a) either with or without 200 mM DTT in the reaction mix. Conversely, purified OxyR from *E. coli* bound the 300 bp *E. coli* target sequence and shifted its mobility progressively as the OxyR concentration increased, but no shift was seen with purified OxyR from *C. diphtheriae* either with or without 200 mM DTT in the reaction mix. These findings show that the purified OxyR proteins from *C. diphtheriae* and *E. coli* exhibit significant differences in their DNA binding specificities.

## Discussion

There are few previous reports of enzymes and regulators involved in responses to oxidative stress in the human pathogen *C. diphtheriae* or the closely related soil bacterium *C. glutamicum*. The *sodA* gene that encodes the manganese-containing superoxide dismutase, an enzyme that scavenges superoxide radicals and protects against superoxide radical-induced oxidative damage, was cloned from *C. glutamicum* and characterized [23]. A previous study from our laboratory showed that insertional inactivation of the diphtheria toxin repressor gene (*dtxR*) in *C. diphtheriae* results in increased susceptibility to growth inhibition and killing following exposure to H_2_O_2_ [31]. In addition, exposure of *C. diphtheriae* to several stressors, including acid, cold, heat, ethanol or SDS, but not exposure to H_2_O_2_, increased transcription of the contiguous *sigB* and *dtxR* genes from the promoter located just upstream from *sigB* [21]. These studies provided initial evidence for metabolic interactions between *dtxR*, the iron-regulon and several non-related stress responses in *C. diphtheriae*.

In the current study, we chose two *C. diphtheriae* reference strains and one *C. glutamicum* reference strain for studies on the role of catalase in resistance to H_2_O_2_ and the role of OxyR, a member of the LysR family of bacterial regulatory proteins, in regulation of catalase (*cat*) gene expression. Published genome sequences for *C. diphtheriae* and *C. glutamicum* have a single gene for catalase [23], [43] and we confirmed production of a single catalase enzyme in *C. diphtheriae* by biochemical analysis (Figs. 2C and 5B.). We showed both by use of *cat::lacZ* reporter plasmids (Figs. 2B and 4B) and by direct quantitative RT-PCR assays of *cat* transcript abundance that OxyR strongly represses *cat* transcription in *C. diphtheriae* by an H_2_O_2_-independent mechanism (Figs. 4C and 4D). Increased production of catalase by Δ*oxyR* mutants of *C. diphtheriae* and *C. glutamicum* conferred a dramatically increased level of resistance to H_2_O_2_ (Figs. 8A and 8B). Transcription of *cat* in *C. diphtheriae* decreased by about 2-fold during transition from the exponential growth phase to stationary phase (Figs. 2B, 4C and 4D). Transcription of *cat* in *C. diphtheriae* was slightly greater under high-iron growth conditions than under low-iron growth conditions, but the difference was statistically significant (Figs. 4C and 4D). We believe it is unlikely that DtxR directly mediates this effect of iron on transcription of *cat*, because the *sigC-cat* intergenic region contains no sequences that closely match the 19 bp consensus sequence for DtxR-binding. Molecular characterization of this effect of iron on *cat* transcription will require additional experiments that are beyond the scope of the current study. We did not identify other environmental signals that regulated *cat* transcription. We found no evidence that exposure of *C. diphtheriae* to H_2_O_2_ stimulates transcription of *cat*. In contrast, transcription of the catalase gene (*katG*) in *E.coli* during exponential growth is activated by OxyR in response to H_2_O_2_, and *katG* transcription during stationary phase is controlled by the starvation-induced sigma factor RpoS [44]. This mechanism of regulating *cat* transcription during stationary phase appears unlikely in *C. diphtheriae* or *C. glutamicum*, because their genomes do not encode *rpoS* [23], [43].

Primer extension analysis showed that a (C) residue located 39 nucleotides upstream from the *cat* ORF is the transcriptional start for the *cat* transcript (Figs. 7A and 7C). A recent study characterized the sigma factors of *C. glutamicum* and features of the specific promoters that they recognize [45]. This analysis of 159 sequences of presumed σ^A^-dependent promoters in *C. glutamicum* revealed a consensus sequence of TANAAT for the −10 hexamer, GN**TA**NAN**T**NG for the extended −10 region, and TTG(A/C)CA for the −35 hexamer in the subset of the corynebacterial promoters for which a −35 sequence was identified. Inspection of the sequence upstream from the *cat* transcriptional start site (Fig. 7C) revealed a putative −10 promoter region ( ***TAAGCT*** , which contains all three of the highly conserved T, A and T residues in the first, second and sixth positions of the −10 consensus sequence). Furthermore, this putative −10 hexamer is properly located with respect to the *cat* transcriptional start site and is also separated by the optimal 17 nucleotides from a putative −35 promoter region ( *TTGCTA* , which matches the consensus −35 sequence at five of six positions). The −10 region has the features of a corynebacterial −10 core hexamer but not of a corynebacterial extended −10 region. In addition, a consensus Shine-Dalgarno ribosome binding site (AGGAGG, labeled **S–D** in Fig. 7C) is present at the proper location upstream from the *cat* ORF. Although future studies will be needed to confirm the functional importance of each of these features for *cat* promoter activity, their presence provides a high degree of confidence that this region does represent the *cat* promoter.

DNaseI footprinting assays identified a 46 bp region protected by OxyR binding that extends from −55 to −10 nucleotides with respect to the +1 nucleotide in the *cat* ORF. This putative OxyR binding site overlaps with the putative −10 hexamer of the *cat* promoter, the start site for the *cat* transcript, and part of the putative ribosome binding site. Binding to this region is fully consistent with repression of *cat* transcription by OxyR, and a previous study in *Pseudomonas putida* showed that the LysR-type regulator CatR represses transcription of the *catR* gene by binding to a region located downstream from the start site of the *catR* transcript and overlaps with the ribosome binding site [46].

The OxyR proteins from *C. diphtheriae* and *C. glutamicum* are members of the LysR family of transcriptional regulators. A characteristic feature of binding sites for LysR-type bacterial regulatory proteins is the presence of one or more highly conserved T-N11-A motifs, each of which may serve as the core for an inverted repeat structure of variable complexity [47]. Examination of the sequence of the OxyR binding site identified by DNaseI footprinting of the *cat* promoter region of *C. diphtheriae* reveals three putative T-N11-A motifs (Fig. 7C). The upstream putative T-N11-A motif (marked with filled diamonds above the conserved T and A residues at positions −53 and −41 with respect to the +1 nucleotide in the *cat* ORF) completely overlaps the putative −10 hexamer of the *cat*. The two partially overlapping downstream putative T-N11-A motifs (marked with filled circles above the conserved T and A residues at positions −36 and −24 and with bold asterisks above the conserved T and A residues at positions −25 and −13) are located between the *cat* transcript start site and the putative ribosome binding site. The horizontal dashed arrows in Fig. 7C mark short palindromic sequences that are associated with, but not always precisely centered on, these putative T-N11-A motifs. Future studies will be required to test directly the functional roles of each of these putative T-N11-A motifs and their associated palindromic sequences in repression of *cat* transcription by *C. diphtheriae* OxyR.

We used complementation tests and gel mobility shift assays to compare the function of the OxyR regulator from *C. diphtheriae* with the well-characterized OxyR regulator from *E. coli*, which was previously shown to act both as a sensor of H_2_O_2_-induced oxidative stress and as transcriptional activator of the catalase gene (*katG*) in *E. coli* [10]. The cloned *oxyR* gene from *C. diphtheriae* (which encodes a protein identical to *C. glutamicum* OxyR) fully complemented the Δ*oxyR* mutation in *C. diphtheriae* or *C. glutamicum* (Figs. 8A and 8B). In contrast, the cloned *E. coli oxyR* gene was shown to be functional in *E. coli* and to direct the production of the identical *E. coli* OxyR protein in *C. diphtheriae* (Fig. 9). These findings indicate that the OxyR proteins from *C. diphtheriae* and *E. coli* have distinct functional specificities, and differences in their ability to interact with OxyR binding sequences from *C. diphtheriae* and *E. coli* were confirmed by gel mobility shift assays (Figs. 10A and 10B).

Bacterial regulatory proteins in the LysR family typically form oligomers. The biologically active form of LysR regulators, including the prototypical OxyR from *E. coli*, is most often tetrameric, although active octamers or dimers have been described in some cases [48], [49]. Fig. S1 shows that primary structure and domain organization of OxyR from *C. diphtheriae* and *C. glutamicum* are homologous with those of other, better characterized, OxyR proteins from a diverse group of bacterial species. We performed limited experiments to examine the oligomeric state of the purified recombinant His6-tagged that we prepared in this study. On a gel filtration chromatography column standardized with several other reference proteins, our *C. diphtheriae* OxyR migrated as a single peak with a mobility consistent with the predicted mass of a trimer (data not shown), but we could not rule out an anomalous mobility because of weak interactions with the gel matrix or other unknown factors. We also made limited but unsuccessful attempts to determine the oligomeric state of *C. diphtheriae* OxyR by several protein cross-linking methods (data not show). Therefore, for this study, we expressed the concentration of *C. diphtheriae* OxyR used in individual experiments as monomer equivalents, and additional future studies will be needed establish definitively the oligomeric state(s) of biologically active *C. diphtheriae* OxyR.

In summary, our results demonstrate that catalase is the effector for defense against H_2_O_2_-induced oxidative stress in *C. diphtheriae* and *C. glutamicum*. They demonstrate that OxyR functions in an H_2_O_2_-independent manner as a repressor of *cat* transcription in *C. diphtheriae*, and they provide initial insights into the organization of the *cat* promoter and the molecular mechanisms for its interaction with OxyR in *C. diphtheriae*. Many questions about the role of OxyR in corynebacteria remain to be examined. For example, does corynebacterial OxyR function as a global transcriptional regulator, and if so what promoters (other than the *cat* promoter) does it repress or activate in corynebacteria? Does corynebacterial OxyR function as a sensor of oxidative stress in corynebacteria, and if so what promoters are preferentially regulated by, and what molecular mechanisms determine the preferential activity of, oxidized or reduced forms of corynebacterial OxyR. Does the regulatory activity of corynebacterial OxyR at some promoters involve interactions with small molecule co-regulators, and if so what is the nature and functional role of such co-regulators. The initial characterization of corynebacterial OxyR reported here should serve as a sound basis for investigating such questions in future studies.

## Supporting Information

Figure S1 **OxyR sequence and organization of the** ***oxyR*** **locus in** ***C. diphtheriae*** **.** A: Alignment of predicted amino acid sequences of OxyR from *Corynebacterium diphtheriae*, *Corynebacterium glutamicum*, *Streptomyces coelicolor*, *Escherichia coli, and Neisseria gonorrhoeae*, assembled using the Clustal W program. The predicted helix-turn-helix motif (HTH) near the amino terminus is indicated by the dotted line; the predicted DNA binding domain lies between the two bent arrows; and the conserved cysteine residues represented by C206 and C215 in OxyR from *C. diphtheriae* or *C. glutamicum* are indicated by asterisks. The numbering of amino acids is shown on the right side. B: Organization of the *oxyR* locus in wild type *C. diphtheriae* C7(β) and in the isogenic C7(β) Δ*oxyR* mutant. The grey arrow shows the orientation of the *oxyR* ORF, and the white arrows show the orientations of the flanking *dirA* ORF (which encodes an iron-repressible polypeptide) and the DIP1422 ORF (which encodes a putative membrane protein). The ORF for the Δ*oxyR* allele, which contains an in-frame deletion, is shorter and is shown to scale in the lower diagram.
(TIF)
