# Supplementary figures and images for: Characterization of OxyR as a Negative Transcriptional Regulator That Represses Catalase Production in Corynebacterium diphtheriae
Source: PLoS One. 2012 Mar 16;7(3):e31709. doi: 10.1371/journal.pone.0031709 (PMC3306370; doi:10.1371/journal.pone.0031709)

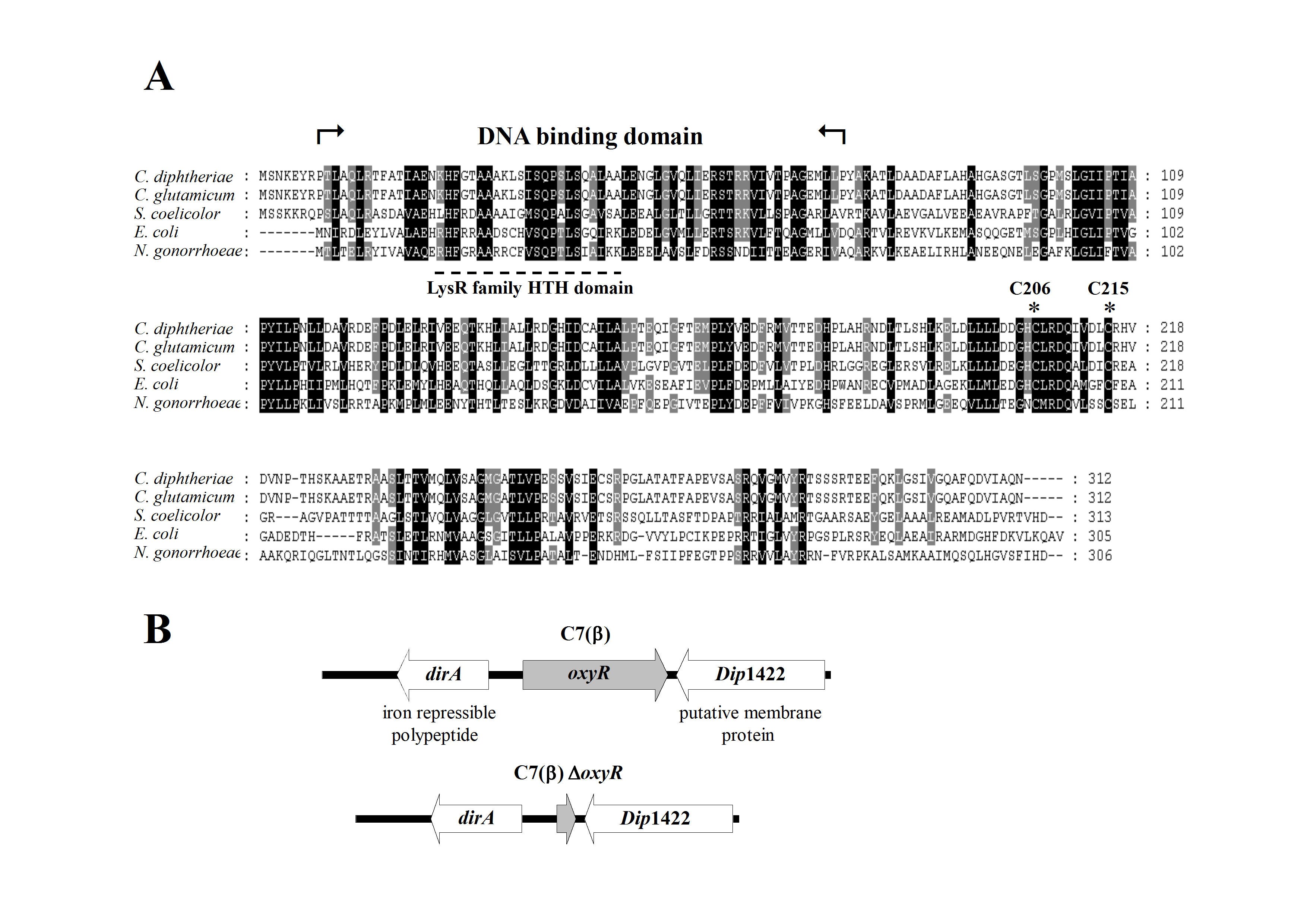

Supplement: Figure S1 — OxyR sequence and organization of the oxyR locus in C. diphtheriae . A: Alignment of predicted amino acid sequences of OxyR from Corynebacterium diphtheriae, Corynebacterium glutamicum, Streptomyces coelicolor, Escherichia coli, and Neisseria gonorrhoeae, assembled using the Clustal W program. The predicted helix-turn-helix motif (HTH) near the amino terminus is indicated by the dotted line; the predicted DNA binding domain lies between the two bent arrows; and the conserved cysteine residues represented by C206 and C215 in OxyR from C. diphtheriae or C. glutamicum are indicated by asterisks. The numbering of amino acids is shown on the right side. B: Organization of the oxyR locus in wild type C. diphtheriae C7(β) and in the isogenic C7(β) ΔoxyR mutant. The grey arrow shows the orientation of the oxyR ORF, and the white arrows show the orientations of the flanking dirA ORF (which encodes an iron-repressible polypeptide) and the DIP1422 ORF (which encodes a putative membrane protein). The ORF for the ΔoxyR allele, which contains an in-frame deletion, is shorter and is shown to scale in the lower diagram. (TIF) [file pone.0031709.s001.tif]
